# Comparative genomics and the nature of placozoan species

**DOI:** 10.1371/journal.pbio.2005359

**Published:** 2018-07-31

**Authors:** Michael Eitel, Warren R. Francis, Frédérique Varoqueaux, Jean Daraspe, Hans-Jürgen Osigus, Stefan Krebs, Sergio Vargas, Helmut Blum, Gray A. Williams, Bernd Schierwater, Gert Wörheide

**Affiliations:** 1 Department of Earth and Environmental Sciences, Paleontology and Geobiology, Ludwig-Maximilians-Universität München, Munich, Germany; 2 Stiftung Tierärztliche Hochschule Hannover, Institut für Tierökologie und Zellbiologie, Ecology and Evolution, Hannover, Germany; 3 Department of Fundamental Neurosciences, University of Lausanne, Lausanne, Switzerland; 4 Electron Microscopy Facility, University of Lausanne, Lausanne, Switzerland; 5 Laboratory for Functional Genome Analysis (LAFUGA), Gene Center, Ludwig-Maximilians-Universität München, Munich, Germany; 6 The Swire Institute of Marine Science and School of Biological Sciences, The University of Hong Kong, Hong Kong; 7 Sackler Institute for Comparative Genomics and Division of Invertebrate Zoology, American Museum of Natural History, New York, New York, United States of America; 8 Department of Ecology & Evolution, Yale University, New Haven, Connecticut, United States of America; 9 GeoBio-Center, Ludwig-Maximilians-Universität München, Munich, Germany; 10 Staatliche Naturwissenschaftliche Sammlungen Bayerns (SNSB)–Bayerische Staatssammlung für Paläontologie und Geologie, Munich, Germany; The Wellcome Trust Sanger Institute, United Kingdom of Great Britain and Northern Ireland

## Abstract

Placozoans are a phylum of nonbilaterian marine animals currently represented by a single described species, *Trichoplax adhaerens*, Schulze 1883. Placozoans arguably show the simplest animal morphology, which is identical among isolates collected worldwide, despite an apparently sizeable genetic diversity within the phylum. Here, we use a comparative genomics approach for a deeper appreciation of the structure and causes of the deeply diverging lineages in the Placozoa. We generated a high-quality draft genome of the genetic lineage H13 isolated from Hong Kong and compared it to the distantly related *T*. *adhaerens*. We uncovered substantial structural differences between the two genomes that point to a deep genomic separation and provide support that adaptation by gene duplication is likely a crucial mechanism in placozoan speciation. We further provide genetic evidence for reproductively isolated species and suggest a genus-level difference of H13 to *T*. *adhaerens*, justifying the designation of H13 as a new species, *Hoilungia hongkongensis* nov. gen., nov. spec., now the second described placozoan species and the first in a new genus. Our multilevel comparative genomics approach is, therefore, likely to prove valuable for species distinctions in other cryptic microscopic animal groups that lack diagnostic morphological characters, such as some nematodes, copepods, rotifers, or mites.

## Author summary

Placozoans are a phylum of tiny (approximately 1 mm) marine animals that are found worldwide in temperate and tropical waters. They are characterized by morphological simplicity, with only a handful of cell types, no neurons, no tissue organization, and even no axial polarity. Since the original description of *Trichoplax adhaerens* 135 years ago, no additional accepted species has been established, leaving the Placozoa as the only animal phylum with only a single formally described species. While classical morphological species identification has failed to reveal further species, single-gene DNA sequence analyses have identified a broad and deep genetic diversity within the Placozoa. To address the significance of this deep genetic diversity in this morphologically uniform phylum, and to better understand its consequences for speciation processes, general biology, and species delimitation in the Placozoa, we sequenced the genome of the placozoan isolate “H13,” a lineage distantly genetically related to *T*. *adhaerens*. Our multilevel genomic comparisons with the *T*. *adhaerens* genome show considerable differences in the general structure of the genome and the makeup and history of various gene families of biological relevance to habitat adaptation. Based on comparative genomics, we here describe the second placozoan species and show that it belongs to a new genus.

## Introduction

**Fig 1:**
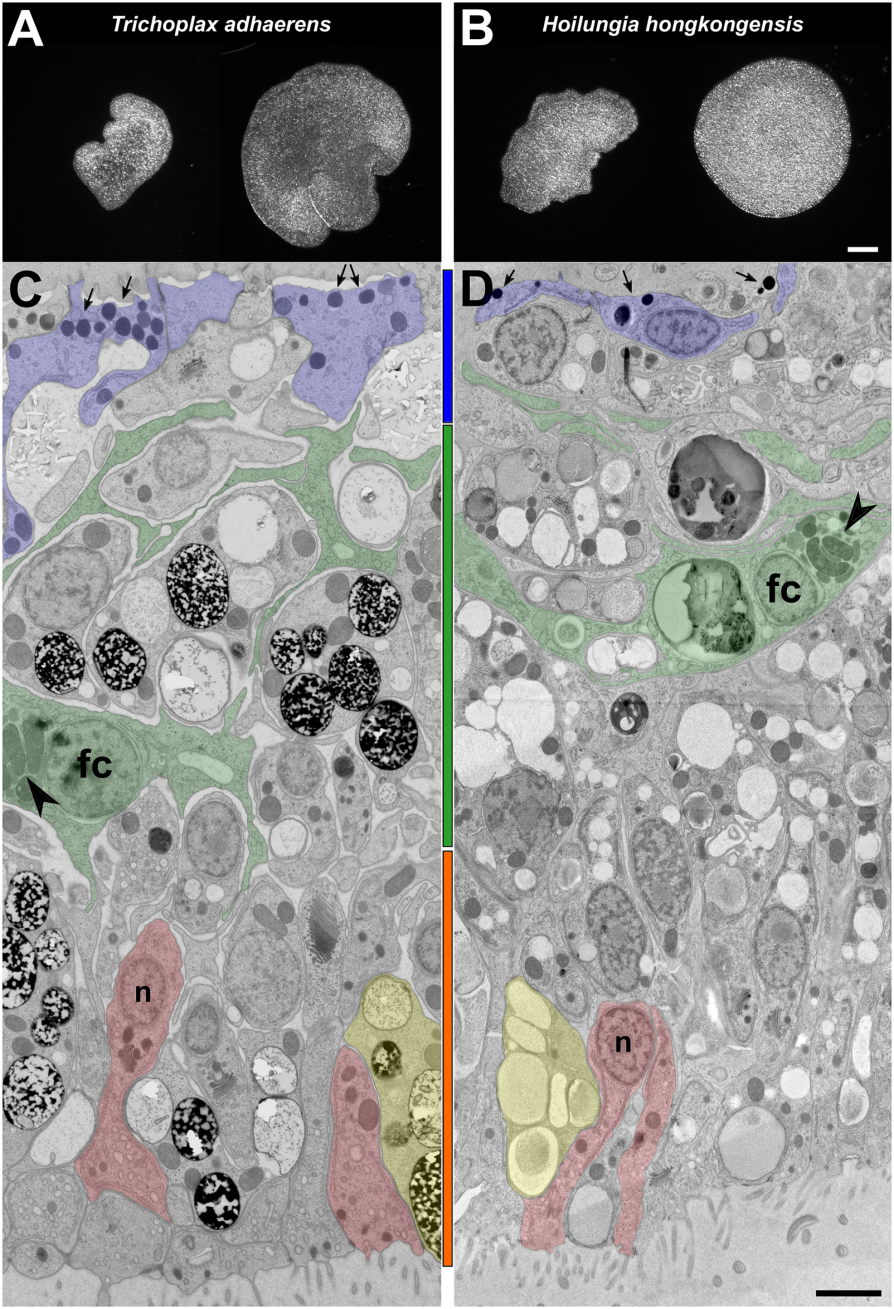
Comparative morphology of *T*. *adhaerens* and *H*. *hongkongensis* nov. gen., nov. spec. Gross morphology and ultrastructure for *T*. *adhaerens* (A, C) and *H*. *hongkongensis* (B, D), respectively. Even within one placozoan clonal lineage, shape plasticity is high, as seen under light microscopy (A, B). At the same time, the internal structure of *T*. *adhaerens* is identical to *H*. *hongkongensis*, as shown in cross sections visualized by transmission electron microscopy (C, D). Both species share the typical placozoan three-layered body plan and the same set of cell types. The upper epithelium (region highlighted with a blue bar between panels C and D) faces the water with monociliated cells (example cell bodies highlighted in light blue). Besides their flat appearance, another characteristic of these cells is the presence of dense granules (arrows) that are typically found toward the upper membrane. The intermediate layer (green bar) consists of a mesh of interconnected nonciliated fiber cells (labeled “fc”; one fiber cell and selected extensions highlighted in light green). Arrowheads mark the mitochondrial complex, one of the defining characters of a fiber cell [9]. Fiber cells are contractile and responsible for the relatively fast shape changes of the animal [7,9]. The lower epithelium (orange bar) is mostly made up of monociliated cylinder cells (two examples marked in light red), whose nucleus (labeled “n”) lies characteristically in the proximal half of the cell body, and lipophil cells (highlighted in yellow) that are rich in large vesicles. The lower epithelium layer is responsible for the ciliated movement of the animal, in addition to feeding [9]. Note the consistent thickness of approximately 20 μm for both individuals. Identified cell types of *H*. *hongkongensis* are further documented in S1 Fig. Scale bar is 100 μm for (A, B) and 2 μm for (C, D).

Placozoans Grell, 1971, are small, benthic marine animals found worldwide in various habitats [1–6]. To date, only a single species has been described, *Trichoplax adhaerens* Schulze 1883. Animals are flat and have a typically disc-like morphology but have the capacity to change shape [7–9]. The lack of symmetry axes, neurons, and defined muscle cells, and the presence of only six morphologically distinguishable somatic cell types ([9,10]; Fig 1, S1 Fig), makes the Placozoa morphologically the most simply organized animals. The prominent placozoan modes of reproduction are asexual, i.e., binary fission and budding [8,9,11–13] that produce genetically identical clones. Sexual reproduction has rarely been observed under laboratory condition [14–19], but both oocytes and sperm cells have been reported [14,17,19], and fertilization, likely coupled with genetic exchange, was confirmed based on structural similarities of the placozoan eggshell with the fertilization membrane of other animal groups [16].

No sexually reproducing individual has ever been reported from the wild. However, the occurrence and success of sexual reproduction in the field have been demonstrated by DNA sequence analyses, since nuclear-encoded marker genes have revealed the occurrence of allele sharing and mixing of heterozygous alleles in a natural placozoan population isolated from a Caribbean habitat [20]. These molecular signatures for genetic exchange prove that sexual reproduction does occur and that the life cycle is completed in the natural environment. However, all efforts to follow the placozoan embryonic development in the laboratory have failed to date. All embryos died at an early stage during development, never reaching beyond the 128-cell stage [19]. The fragmentation of the nucleus in the zygote [21] was previously suggested as the reason for the termination of development, although this has been questioned [19]. This ambiguity and scarcity of information has, therefore, left us with a large knowledge gap regarding the life history of the Placozoa and has resulted in speculations of the existence of a missing life stage (compare [22]).

The genome of the diploid *T*. *adhaerens* was sequenced previously [22], revealing that this morphologically very simple animal harbors a rich repertoire of gene families [22]. These families are known from bilaterian animals and are typically associated with a considerable cell type diversity, a complex body plan, developmental processes, and behavioral responses to external stimuli [10,23–31]. Additionally, single-gene molecular phylogenetics have identified a sizeable cryptic diversity within placozoans collected worldwide; but while their gross morphology is highly plastic, morphologically, all isolates fit the description of *T*. *adhaerens* [32] (Fig 1). The high intraspecific shape variability, coupled with an ultraconserved internal structure (Fig 1, S1 Fig), does not allow the establishment of reliable diagnostic morphological characters in the Placozoa, hindering attempts to characterize their diversity.

While these single-marker studies provided clear indications that additional species may be uncovered in the Placozoa, two fundamental questions remain: how different are placozoans at the nuclear genome level, and what can we learn from comparative genomics about the evolution and diversity of placozoans? To address these questions, we generated a high-quality draft genome of a placozoan lineage that is genetically distantly related to *T*. *adhaerens* [3,5] and performed a multilevel comparison, including genome synteny, gene clustering, gene ontology (GO) term enrichment, allele sharing, and cross-phylum comparative distance analyses. This approach, together with the morphological characterization of the lineage H13, allowed us to assign a taxonomic status to morphologically cryptic taxa and led to the establishment of the second placozoan species in a new placozoan genus.

## Results and discussion

### Adding a new placozoan genome and improving the *T*. *adhaerens* genome annotation

Based on mitochondrial 16S ribosomal DNA (rDNA) analyses, the genetic lineage H13 is among the most distantly related haplotype to *T*. *adhaerens* (lineage H1) [5], whose nuclear genome has been sequenced previously [22]. We hypothesized that the substantial 16S rDNA divergence might also be reflected on the whole-genome scale and, therefore, targeted H13 for nuclear genome sequencing. To assemble the genome of H13—a new species described here, called *H*. *hongkongensis* nov. gen., nov. spec. (Fig 1, S1 Fig; see species description in Material and methods; Tables 1 and 2)—we generated 24 Gb of paired-end reads and 320 Mb of Moleculo (Illumina Artificial Long Synthetic) reads. Our final, highly complete 87-megabase assembly contained 669 high-quality and contamination-filtered contigs with an N50 of 407 kb (S1 Table; S2–S4 Figs), 7 megabases smaller than the *T*. *adhaerens* contig assembly. The overall calculated genome heterozygosity (based on single-nucleotide polymorphism [SNP] counts, see S2 Table) was 1.6%, which is moderate for a marine animal but about average when compared to arthropods and high in comparison to terrestrial chordates [33]. This value cannot be compared to *T*. *adhaerens* because of the low genome coverage of the latter, which does not allow haplotype phasing.

**Table 1:** Molecular 16S rDNA diagnostics for the genera *Hoilungia* and *Trichoplax*.

alignment position	1,877	2,017	2,026	2,046
*Hoilungia*	T	A	T	A
*Trichoplax*	A	G	G	G

**Table 2:** Molecular 16S rDNA diagnostics for *H*. *hongkongensis*.

alignment position	1,859	1,886	2,031	2,102	2,105	2,110	2,111	2,114	2,128	2,129	2,144	2,163	2,179	2,196
*H*. *hongkongensis* (16S clade V)	A	T	C	A	A	A	A	A	T	T	T	A	A	T
16S clades III, IV,VI,VII	T	A	A	G	C	G	G	G	C	C	C	G	T	C

We annotated the genome with a combination of 15.3 Gb of RNA-Seq and *ab initio* methods to yield 12,010 genes (S1 Table, S1 & S2 Data). A high percentage of raw reads mapped back to the genome (S3 Table), and between 90.8%–95.3% of the 978 genes in the BUSCO v3 Metazoa dataset were identified in the transcriptome and the *ab initio* gene models, respectively (S4 Table). Together, this suggests an almost complete assembly and annotation, in which 96.5% of the genes in the *H*. *hongkongensis* genome were expressed in what are commonly considered adult animals. In our gene set, *H*. *hongkongensis* had 490 more genes than the 11,520 genes reported in the original *T*. *adhaerens* annotation from 2008 [22]. We reannotated *T*. *adhaerens* with AUGUSTUS and found an additional 1,001 proteins and also managed to complete formerly partial proteins (for *T*. *adhaerens* Blast2GO protein annotations see S3 Data). This approach added 4.4 Mb of exons to the *T*. *adhaerens* annotation, an increase of 28% of exonic base pairs to the original annotation. The new *T*. *adhaerens* annotation now has 511 more genes than *H*. *hongkongensis*, which accounts for some portion of the size difference between the two genomes.

### Genomic rearrangements are commonplace

**Fig 2:**
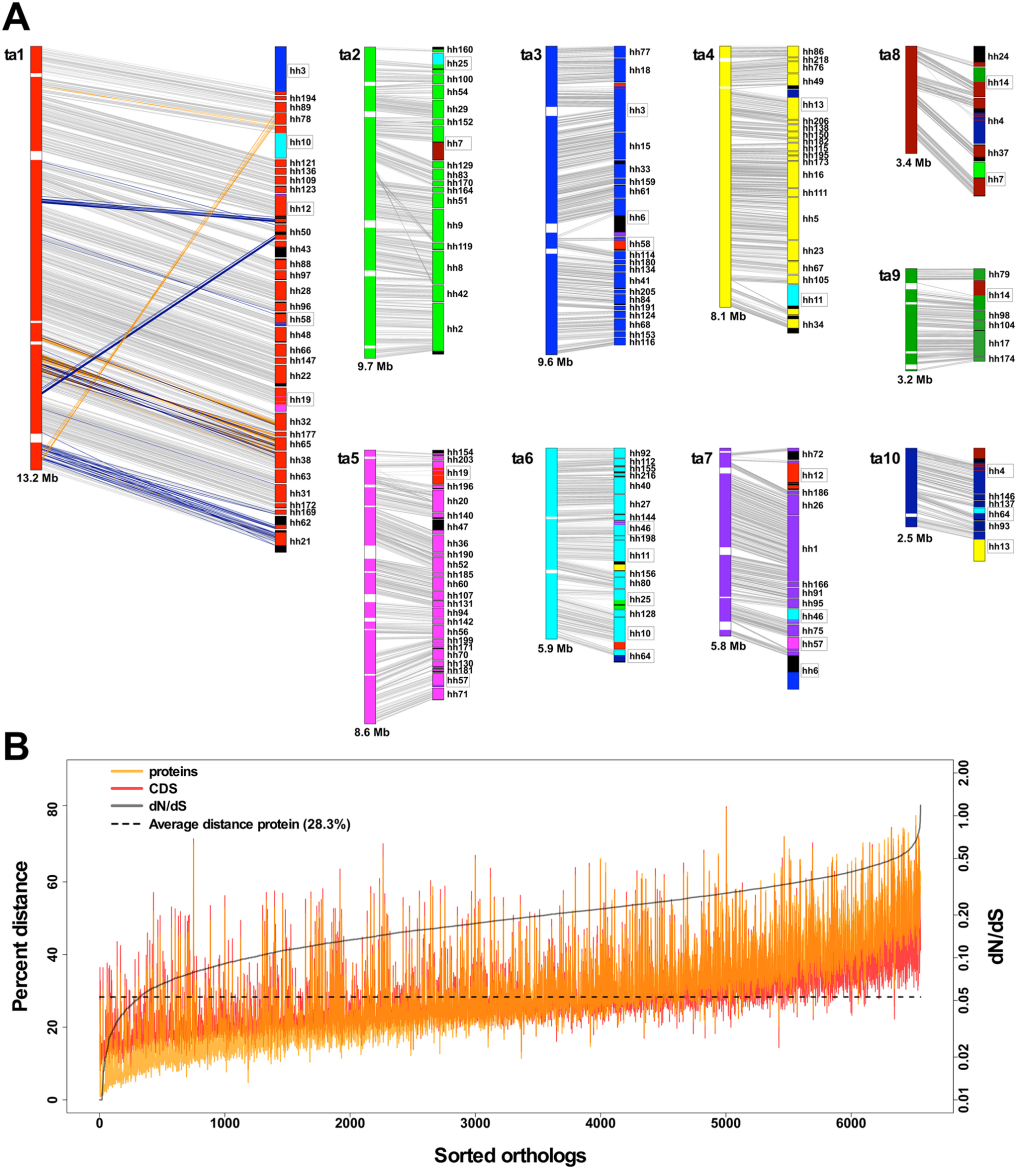
Extensive genomic structural differences and significant genetic divergence between *T*. *adhaerens* and *H*. *hongkongensis*. (A) Scaled schematic drawings of the 10 longest *T*. *adhaerens* scaffolds on the left (ta1–ta10) and matching *H*. *hongkongensis* contigs on the right. While a general macrosynteny between the two placozoan species is present (gray lines), 25% of the genes are translocated (blue lines) or inverted (orange lines) relative to the order of the respective *T*. *adhaerens* scaffold (illustrated for ta1). Often, entire gene blocks are translocated (different colors in boxed *H*. *hongkongensis* contigs). Black stretches mark genomic regions not matching any of the 10 *T*. *adhaerens* scaffolds, while white stretches mark gaps in the *T*. *adhaerens* scaffolds. (B) Pairwise protein and CDS distances for 6,554 one-to-one orthologous genes. A significant fraction of orthologs have larger protein than CDS distance, but only three of these are, in fact, positively selected (reflected by dN/dS ratios > 1, gray line). Orthologs are sorted by increasing dN/dS. Calculated distances can be found in the *H*. *hongkongensis* data repository at https://bitbucket.org/molpalmuc/hoilungia-genome/src/master/orthologs/. CDS, coding sequence; dN/dS, nonsynonymous to synonymous nucleotide substitutions.

Moleculo reads also enabled us to assemble very large reference contigs, the longest being over 2 Mb. We compared the organization of genes in *H*. *hongkongensis* to the 10 longest scaffolds in the *T*. *adhaerens* genome (size range 2.4–13.2 Mb; accounting for 66% of the *T*. *adhaerens* assembly). We found 144 contigs >100 kb from *H*. *hongkongensis* that aligned to these 10 scaffolds, accounting for 69% of the *H*. *hongkongensis* assembly (Fig 2A). Mean gene collinearity (i.e., the same genes in the same direction) in this reduced genome representation was in the range of 69.5% to 78.8% (mean 73.6% ± 5.5%; see S5 Table). The mean number of genes per syntenic block was 33.8 (±25.2) in the reduced set and 33.9 (±24.7) when comparing full genomes (S5 Fig), which indicates that the reduced set is representative for both complete genomes.

Although much of the gene order is conserved between the two species, we counted 2,101 genes (out of the 8,260 genes in the 10 scaffolds) that were inverted or translocated within the same scaffold relative to the order in the *T*. *adhaerens* scaffolds. These numbers seem low when compared to the fast-evolving bilaterian genus *Drosophila* [34,35] or *Caenorhabditis* [36], but they are in the range of rearrangements found between mouse and human [37]. Comparison to Bilateria, however, might be misleading (see also results on genetic distances below), and genome rearrangement events might be more favored in some bilaterian taxa because of inherent genomic traits such as transposon-induced rearrangement hotspots [38]. Nonetheless, the high percentage of rearrangements between *T*. *adhaerens* and *H*. *hongkongensis* is clear evidence for a deep genetic separation of both lineages.

### Sequence divergence analyses identify unexpectedly high genetic distances between *H*. *hongkongensis* and *T*. *adhaerens*

To estimate how divergent the two placozoan genomes are at the sequence level, we calculated genetic distances for 6,554 one-to-one orthologs. Between *H*. *hongkongensis* and *T*. *adhaerens*, genetic distances ranged from 0.9% to 80.1% (mean 28.3% ± 12.9%) for proteins and 7.4% to 80.7% (mean 28.5% ± 9.9%) for coding sequences (CDSs), respectively (Fig 2B). To assess if certain genes are under positive (diversifying) selection, indicative of functional evolution, we calculated the ratio of nonsynonymous to synonymous nucleotide substitutions (dN/dS ratio [39]) for each *H*. *hongkongensis* and *T*. *adhaerens* one-to-one ortholog pair. Results show that most orthologs (97%) are under strong purifying selection (dN/dS < 0.5). One might hypothesize that strong purifying selection pressure is the reason for the phenotypic stasis we see in modern placozoans. However, more placozoan genomes in the phylum are clearly needed to test this hypothesis. Despite this strong tendency toward purifying selection, a high proportion of orthologs (46%) showed larger protein distance than CDS distance and, therefore, an accumulation of double or triple mutations in already mutated codons, which led to amino acid substitutions (S6 Fig).

Only 3 of the 6,554 one-to-one orthologs had dN/dS ratios slightly >1, indicating positive selection (S7 Data; see S6 Fig for an estimate of mutation saturation in codons). One of these seems placozoan specific, since it could not be annotated because of missing UniProt BLAST hits and InterPro domains, respectively. For the second, GO annotation and InterPro IDs indicate a role in telomere maintenance. The third positively selected gene (CYP11A1) is putatively a cholesterol side-chain cleavage enzyme acting in the mitochondrion.

The roughly 4x coverage of the genome with long Moleculo reads (N50 of 5.4 kb) allowed the assembly of large haplocontigs (i.e., contigs representing both haplotypes of the genome). This phasing information for large parts of the genome facilitated the isolation of 2,870 one-to-one orthologs with both full-length alleles after a highly stringent filtering procedure. Only by using the phasing information we were able to show that many orthologs with high allelic variation in *H*. *hongkongensis* were also profoundly different between the species (S7 Fig). This indicates that genetic sequence adaptation already takes place at the population level and is further magnified between species in the same genes.

### Adaptation by gene duplication is one key mechanism for speciation in the Placozoa

The Markov cluster (MCL) analysis identified 6,644 true one-to-one orthologs (for an overview of ortholog categories, see Material and methods and [40]) for both placozoan species (55% of all proteins in *H*. *hongkongensis* and 53% in *T*. *adhaerens*, respectively) (S8 Fig). A fraction of 465 (3.8%) *H*. *hongkongensis* and 1,036 (8.3%) *T*. *adhaerens* proteins, respectively, did not have reciprocal BLAST hits. The difference in the non-BLAST hits almost perfectly matches the differences in total gene numbers, which is probably an indication that genes without a homolog in *H*. *hongkongensis* account at least partially for the slightly higher gene number in *T*. *adhaerens*. A high proportion of proteins had BLAST hits to the UniProt database, and only 15.4% (1,859) and 19.0% (2,384) of *H*. *hongkongensis* and *T*. *adhaerens* proteins, respectively, did not have BLAST hits to metazoans included in UniProt.

Placozoan-specific duplications constitute a significant proportion of both proteomes, with 3,943 (32.8%) co-orthologs in *H*. *hongkongensis* and 3,484 (27.8%) in *T*. *adhaerens*. The enrichment analyses for the proteins in each non-BLAST-hit bin identified unique GO terms in all three GO categories among the first five most significantly enriched GO terms (S4 & S5 Data). The same applies to one-to-many and many-to-one co-orthologs in both species.

The enrichment analyses further indicate that both placozoan species have multiple co-orthologs associated with G-protein-coupled receptor (GPCR) signaling. A rich repertoire of GPCRs has been identified in *T*. *adhaerens* [22], but here, we were able to identify independent GPCR duplications in *H*. *hongkongensis* and *T*. *adhaerens*, respectively (S6 Data). Furthermore, we identified multiple enriched GO terms related to synaptic activity in all co-ortholog categories (S5 Data) and both placozoan species. This points to a plethora of independent duplication events in gene families related to sensory capacities. Despite lacking neurons (based on traditional morphological classifications), *T*. *adhaerens* has previously been shown to stain positive for FMRFamide [10,41] and recently even to change behavior when exposed to physiologically relevant levels of neuropeptides [31].

Based on the identification of vast and independent gene family expansions in both placozoans, we propose that adaptation in the Placozoa, ultimately leading to speciation, is coupled with independent gene duplications as suggested, for example, for bacteria, yeast, plants, and other animals (compare [42–45]). *H*. *hongkongensis* was isolated from a stream running through a mangrove with rapid drops in salinity and temperature, especially during heavy rainfall in the summer. We hypothesize that the presence of multiple divergent copies of genes involved in various processes, such as behavior and metabolism (compare [42,43]), in addition to a situation-dependent expressional fine-tuning of these copies was necessary for adaptation to this habitat and would facilitate speciation. We furthermore propose that the presence of multiple copies of genes and their expression does not affect the phenotype but instead provides a genetic toolkit for gradual physiological responses to (changes in) the environment.

### Allele sharing analyses identify reproductive isolation between placozoan clades

All internal Linnaean ranks within the Placozoa are, as yet, undefined [5]. Despite efforts to identify them, reliable diagnostic morphological characters, commonly used for defining animal species, are lacking in the Placozoa [46]. Thus, all present taxonomic definitions in the phylum must solely rely on diagnostic molecular characters. In other taxonomic groups (e.g., bacteria and archaea [47], protists [48,49], and fungi [50]), purely sequence-based approaches and working models for the distinction of taxa have been proposed and are generally well established and widely accepted [51]. In animals, such methods (which may be based on distances, on trees, or on allele sharing; [52]) are currently under development and have been used in rare cases to identify and describe cryptic species [53].

**Fig 3:**
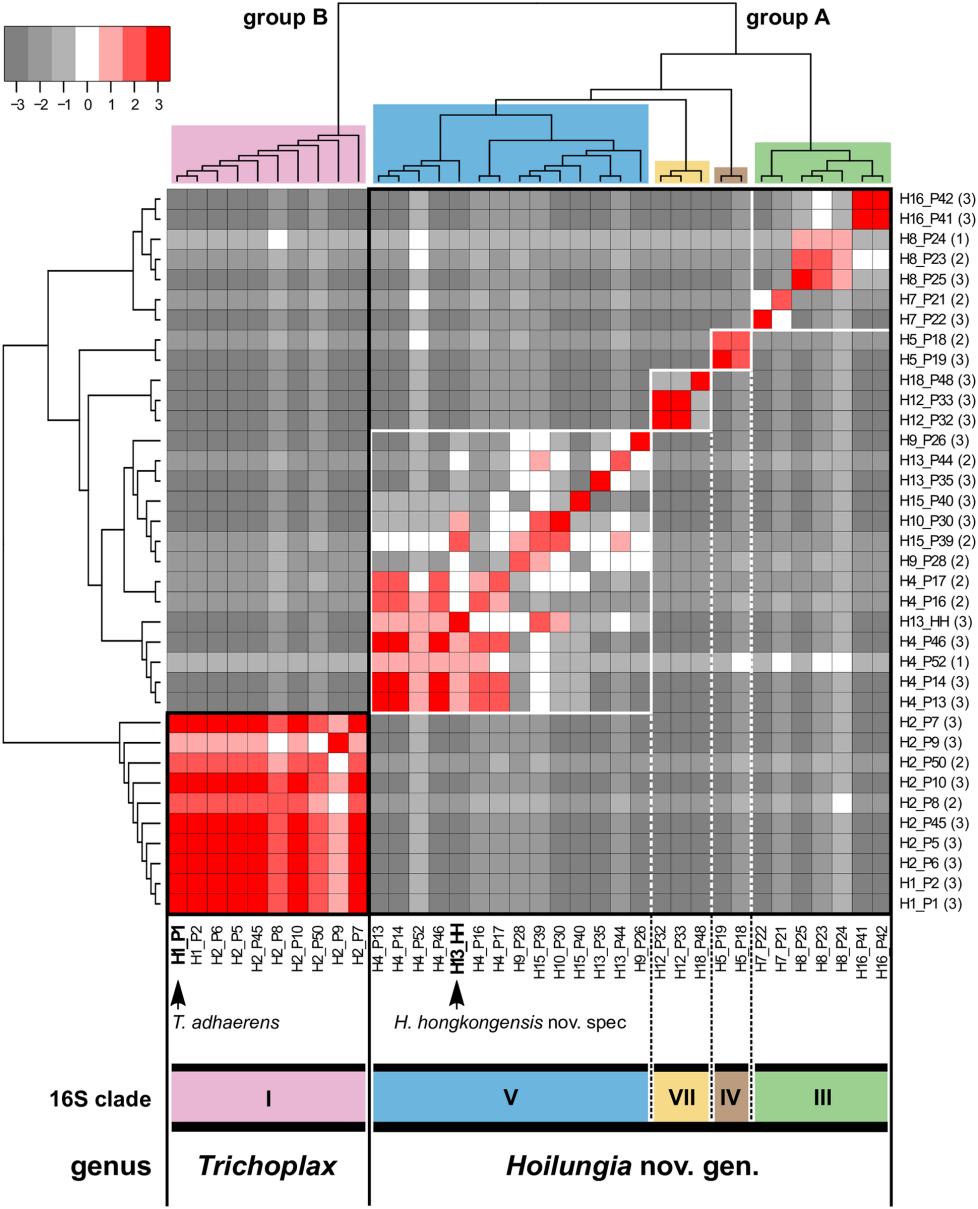
CM highlighting reproductive isolation between clades and a split into two genera. The CM for three nuclear-encoded ribosomal proteins (*rpl9*, *rpl32*, and *rpp1*) was generated by calculating (for each pair of isolates) the number of markers supporting their conspecificity in haploweb analyses (i.e., different individuals can be assigned to one species by shared alleles) minus the number of markers supporting the premise that they belong to different species. The CM was visualized as a heat map with different colors representing various amounts of shared alleles from −3 (no shared alleles) to +3 (3 shared alleles). Higher scores (red), therefore, indicate conspecific isolates, while gray tones support reproductive isolation, i.e., separate biological species. The number of sequenced markers per isolate is given in brackets beside the isolate (see S6 Table for details on isolates). The CM shows that allele sharing can occur between haplotypes within but never between clades. This is the first evidence for reproductive isolation between placozoan clades and the first molecular support for the existence of biological species in the Placozoa. The CM furthermore supports the phylogenetic split between *Trichoplax* (clade I; note: no data available for clade II) and the new placozoan genus *Hoilungia* (clades III–VII), as shown in the dendrogram on top of the heatmap. These clades are consistent with those recovered from analyses of the mitochondrial ribosomal large subunit (16S) [5] and compensatory base changes in the ITS2 [55]. Data underlying this figure can be found at https://bitbucket.org/molpalmuc/hoilungia-genome/src/master/reproductive_isolation/. CM, conspecificity matrix; ITS2, internal transcribed spacer 2.

In a first step to converting the identified genomic differences into a taxonomically meaningful system, we studied reproductive isolation by addressing allele sharing within placozoan isolates from different localities. To identify reproductive isolation, a conspecificity matrix (CM) was generated [54]. The CM was based on three nuclear genes encoding ribosomal proteins and clearly identified reproductive isolation between placozoan clades (Fig 3). This approach extends a previous study that has uncovered sexual reproduction only within one placozoan haplotype (H8) [20] and provides clear evidence that the previously established placozoan clades (based on 16S genotyping) are reproductively isolated biological species.

### Cross-phylum comparative distance analyses allows the establishment of a new genus in the Placozoa

**Fig 4:**
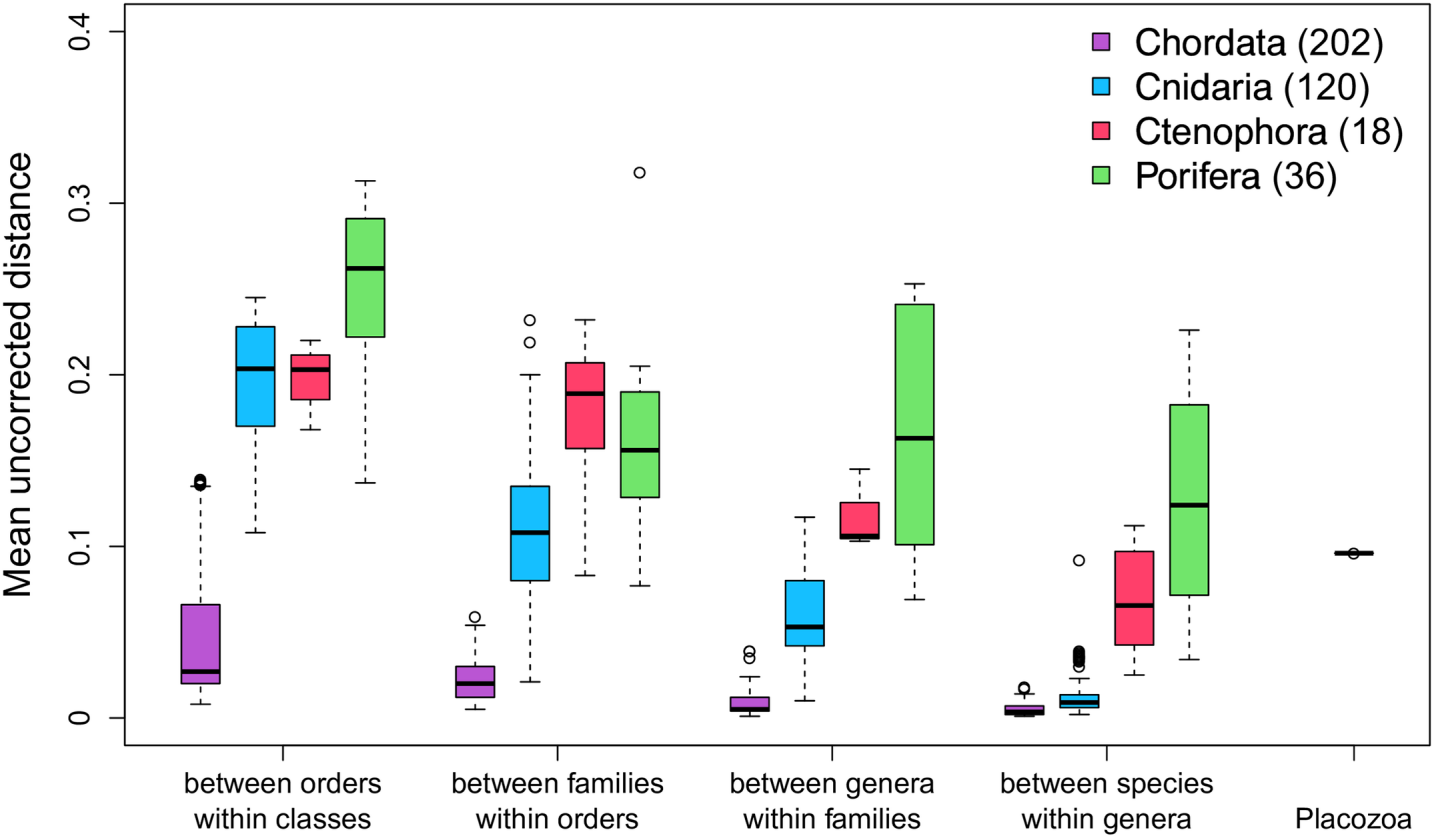
Calculated uncorrected pairwise genetic distances for 212 concatenated nuclear-encoded proteins (dataset 1). Mean group distances for different taxonomic ranks in three nonbilaterian phyla (Cnidaria, Ctenophora, and Porifera) and the bilaterian phylum Chordata. The interspecific protein distance of 9.6% between *H*. *hongkongensis* and *T*. *adhaerens* (right) is comparable to mean group distances between genera within families in the Ctenophora. With respect to the Cnidaria, the placozoan distance is even comparable to the mean group distance between families within orders. Measured distances for families within orders in Ctenophora and genera within families in Porifera indicate that classical morphological taxonomies are incongruent with the calculated genetic distances in these two phyla (see also S9–S14 Figs). The internal phylogeny of these two phyla appears to be in urgent need of further reevaluation with the inclusion of molecular data (compare [57–60]). Measured distances in chordates fall way below distances calculated for the nonbilaterian taxa for all levels of comparison. Numbers in brackets are total taxa in the final matrix of 212 concatenated proteins. For calculated distances, see S8 Data.

We have shown that biological species exist in the Placozoa. Previous studies have furthermore provided first indications for the existence of deeper differences between placozoan lineages [1,3], with as-yet-unknown correspondence to, for example, the Linnaean ranks of genus, family, order, and class. However, these observed deeper divergences were based on single marker genes only, and no diagnostic morphological traits could be identified to establish a firm, higher-level, systematic framework in the Placozoa. To further estimate the level of taxonomic relatedness between *T*. *adhaerens* and the new placozoan species *H*. *hongkongensis* (strain H13), and in an attempt to initiate a higher-level taxonomic system for the Placozoa, we performed cross-phylum multimarker sequence divergence analyses. To do so, we compared the variation between the two placozoans to variation within the other three nonbilaterian phyla, Cnidaria, Ctenophora, and Porifera (compare [1]), as well as the bilaterian phylum Chordata. Marker sets included a nuclear protein set of 212 concatenated proteins (dataset 1, a taxon-extended matrix from [56]; S7–S9 Tables; see Fig 4) as well as 5 selected genes with different substitution rates (S9–S14 Figs), all commonly used for DNA barcoding and molecular systematics.

Across individual markers, it appears that the phylogenetic ranks are most robust in the Cnidaria, in which the partitioning of molecular variation matches the established taxonomy, in that Linnaean ranks consistently correspond to the greater distance between groups (Fig 4; S9–S14 Figs). The same is true for the Chordata, which was included in our distance calculations for the 212 nuclear protein set as an example of a bilaterian phylum with a high taxonomic coverage (many genomes are available for this group). However, distances in chordates are, in general, much lower when compared to the overall more similar nonbilaterian phyla. This indicates that (i) genetic distances and corresponding Linnaean rank assignments in Chordata cannot be compared to nonbilaterian lineages and (ii) that comparisons among nonbilaterians are better suited to guide taxonomic ranking of the two placozoan species. We consequently used genetic distances in the Cnidaria as an approximation and comparative guideline for the higher systematic categorization of the new placozoan species.

Genetic distances between *H*. *hongkongensis* and *T*. *adhaerens* were higher than those for the Cnidaria in five of the six marker sets at the generic level but lower at the family level for all markers (S14 Fig, S10 Table), which, cautiously interpreted, supports genus-level genetic differences between the two placozoans.

A clear split of the Placozoa in the molecular groups “A” and “B” was previously shown by the rearrangement pattern of mitochondrial genomes [61] and compensatory base changes in the internal transcribed spacer 2 (ITS2) [55]. The conspecificity analysis, the high amount of genomic rearrangement, and the large-scale independent gene duplication history, as well as the genetic distances in six independent datasets, strongly support this split (Fig 3). Since clades were identified as the primary taxonomic units—i.e., biological species—these two previously identified higher-level placozoan “groups” consequently represent at least the genus level in the Linnaean hierarchical system. We therefore establish the new genus *Hoilungia* for the former group “A” (clades III–VII), which is, so far, the single sister genus to *Trichoplax* (former group “B”; clades I and II).

Future research efforts focusing on genome sequencing of additional placozoan clades/species will likely help to establish a broader and more detailed systematic framework for the Placozoa and provide further insights into the mechanisms and driving forces of speciation in this enigmatic marine phylum.

### The *H*. *hongkongensis* genome adds support to the phylogenetic placement of the Placozoa in the animal tree of life

Recent discussions about the phylogenetic position of placozoans have largely been based on the *T*. *adhaerens* genome. A better sampling of placozoan genomic diversity is, however, needed [62] to address their placement in the metazoan tree of life. In this context, it is important to first assess if adding another placozoan genus would break up the long placozoan branch. The inclusion of a single representative of a clade with a very long terminal branch, or fast-evolving taxa that can have random amino acid sequence similarities, may result in erroneous groupings in a phylogeny (so-called “long-branch attraction artefacts”) [63,64]. To address these questions, we generated a highly (taxa) condensed version of the full protein matrix from Cannon and colleagues [56] (termed dataset 2; with less than 11% missing characters and 194 genes). We additionally created a Dayhoff 6-state recoded matrix [65,66] of this second set to reduce amino acid compositional heterogeneity, which is also known to be a source of phylogenetic error [67,68]. Phylogenetic analyses were performed on these two matrices (protein and Dayhoff-6 recoded), using the site-heterogeneous CAT-GTR model in PhyloBayes-MPI [69] and using the site-homogenous GTR model both in Phylobayes-MPI and RAxML (RAxML, protein only) [70], as well as the LG model in RAxML (protein only). The resulting trees (S15–S20 Figs) of the highly dense gene matrix (S21 Fig) suggest a sister group relationship of the Placozoa to a Cnidaria + Bilateria clade with both CAT-GTR (Protein, Dayhoff-6 recoded, S15–S17 Figs) and GTR models (Protein, S18 Fig) in PhyloBayes, or these relationships are unresolved (RAxML, protein, both GTR, S19 Fig, and LG, S20 Fig). This is in agreement with some previous findings [56,64,71–74] and with recent studies using a large gene set and intense quality controls [64] as well as improved modeling of compositional heterogeneity [68]. In addition, the sister group relationship of the Placozoa to the Cnidaria + Bilateria clade is corroborated by independent data—namely, the analysis of metazoan genome gene content [73,75,76].

## Material and methods

### Formal taxonomic diagnosis

Phylum: Placozoa, Grell 1971 [77]

Type Family: Trichoplacidae, Bütschli and Hatschek 1905 [78], synonymized with “Trichoplaciden” (in German original), Haeckel 1896 [79].

Diagnosis: We assign all currently known 19 placozoan genetic lineages (16S haplotypes H1-H19; [5]) to the Trichoplacidae. The description of *T*. *adhaerens* Schulze 1883 applies to all.

Type Genus: *Hoilungia*, nov. gen., Eitel, Schierwater, and Wörheide

*Hoilungia* is the second genus of the family Trichoplacidae.

Etymology: *Hoilungia*, pseudo-Latinized from “Hoi Lung,” Cantonese, meaning “sea dragon,” which is based on the shape-shifting dragon king in Chinese mythology.

Diagnosis: Gross and fine morphology appear similar among all placozoans studied to date. We therefore use molecular diagnostics to define Linnaean ranks. Among all tested markers, the mitochondrial large ribosomal subunit 16S rDNA appears to be the most variable among placozoans and other nonbilaterian phyla, and the mean pairwise distance is closest to that calculated for the nuclear dataset in most cases (S14 Fig). This marker also best mirrored classical taxonomy in the Porifera and Cnidaria (S11 Fig; in Ctenophora, 16S rDNA is highly derived and hard to identify [80]). According to these data, molecular diagnostics based on differences in the 16S rDNA appear to be suitable for current and future designation of species in the Placozoa, which is in agreement with previous results [3]. Diagnostics are here, therefore, defined by nucleotide substitutions in the 16S rDNA. Full-length 16S rDNA sequences of *T*. *adhaerens* and *H*. *hongkongensis* (clonal strain “M2RS3-2”), as well as for the undescribed Placozoa sp. H4 and sp. H8, were aligned with MAFFT v7.273 [81] using the GINSI option and otherwise default settings. Ambiguously aligned 5′ and 3′ sequence ends were removed. To this alignment, we added all currently available placozoan 16S haplotype sequences [5] using MAFFT [added option:—add]. The final alignment contained all 19 placozoan haplotypes and had a length of 2,551 nucleotides (including gaps). The region for identification of diagnostic nucleotides was restricted to a part of the 16S alignment that was previously shown to be suitable and sufficient for molecular haplotype discrimination [1,3,5]. We furthermore restricted the identification of diagnostics to stem regions of this rDNA to omit uncertainties in future taxonomic assignment due to ambiguously aligned loop regions. To identify molecular diagnostics for the genus *Hoilungia*, we screened for molecular synapomorphies (nucleotide exchanges) within the placozoan 16S group “A” (clades III–VII; [5,61]) versus group “B” (clades I and II).

Molecular diagnostics for *Hoilungia* and *Trichoplax* are summarized in Table 1.

Type species: *H*. *hongkongensis*, nov. spec., Eitel, Schierwater, and Wörheide.

Diagnosis: To identify molecular species diagnostics, we determined unique substitutions (based on the alignment used for genus diagnostics before) for *H*. *hongkongensis* (clade V) in comparison to the other *Hoilungia* clades (III, IV, VI, and VII).

Molecular diagnostics for *H*. *hongkongensis* are summarized in Table 2.

Type locality: A single specimen of *H*. *hongkongensis* (clonal strain “M2RS3-2”) was isolated in the Ho Chung River close to a small mangrove at Heung Chung village, Hong Kong (22.352728N 114.251733E), on June 6, 2012.

Type specimen: One specimen of *H*. *hongkongensis* (clonal strain “M2RS3-2”) has been mounted and deposited at the Bayerische Staatssammlung für Paläontologie und Geologie in München, Germany, under voucher number SNSB-BSPG.GW30216. Clonal individuals have been stored in ethanol as paratypes under voucher number SNSB-BSPG.GW30217 in addition to a DNA extraction under voucher number SNSB-BSPG.GW30218.

Etymology: *hongkongensis*, from “Hong Kong,” and “-ensis,” Latin, suffix referring to place of origin, as specimens are at present endemic to Hong Kong. The full name “*Hoilungia hongkongensis*” thus means “Hong Kong sea dragon.”

### Animal material

Two strains were used for this project: The “M2RS3-2” strain was used for the DNA sequencing (the “DNA strain”) and the “M153E-2” strain (the “RNA strain”) for the transcriptome. Both strains descend from a single placozoan individual each, which was isolated from mangroves/mangrove associates at two different sites in Hong Kong (SAR, China). The DNA strain was isolated from a dead mussel shell collected in the Ho Chung River close to a small mangrove at Heung Chung village (22.352728N 114.251733E) on June 6, 2012. The habitat undergoes daily changes in salinity, and on the day of collection, the salinity was 20 psu. The RNA strain was isolated from collection traps (for details on slide sampling, see [82]) connected to mangrove associates (*Hibiscus* sp.) and high shore mangrove (*Excoecaria* sp.) trees at Tai Tam Tuk (22.244708N 114.221978E) on March 30, 2012. Both clonal cultures were cultured in 14 cm glass Petri dishes as described [19], with a pure *Pyrenomonas helgolandii* algae culture (strain ID 28.87, Culture Collection of Algae, Georg-August-Universität Göttingen). The two different strains were used for DNA and RNA sequencing, respectively, to identify polymorphisms in these strains living in the same habitat but at two hydrogeographically distinct sampling sites (northeast versus southeast Hong Kong).

### Morphological analyses

Animals were transferred in 20% BSA in artificial seawater, high-pressure frozen in a Wohlwend HPF Compact 02, and stored in liquid nitrogen. Samples were processed from −90 °C to room temperature for Epon embedding in a Leica AFS unit as follows: they were fixed and contrasted in 0.1% tannic acid in acetone for 24 h and washed 4 times for 15 min in acetone; samples were then incubated in 2% Osmium tetroxide in acetone while the temperature was increased stepwise to −40 °C within the next 23 h; samples were then washed and progressively infiltrated in Epon:acetone mixes (1:2, 2:1) and pure Epon while temperature was further raised from −40 °C to room temperature over 6 h. They were then polymerized in Epon. Seventy-nm ultrathin sections were cut on a Leica Ultracut and picked up on a copper slot grid 2 × 1 mm coated with a polystyrene film. Sections were poststained with uranyl acetate 2% in distilled water for 10 min, rinsed several times with distilled water followed by Reynolds lead citrate in distilled water for 10 min, and rinsed several times with distilled water. Micrographs were taken with a Transmission Electron Microscope Philips CM100 at an acceleration voltage of 80 kV with a TVIPS TemCam-F416 digital camera.

### Genome sequencing and assembly

#### Short-read sequencing

DNA was isolated as described [83] from roughly 1,000 healthy growing and clonally dividing individuals. Genomic DNA (150 ng) was used to prepare an Illumina-compatible paired-end library with a nominal insert size of 250 bp. All steps were done using the reagents from the Accel DNA 1S library preparation kit (Swift Biosciences, Ann Arbor, United States of America) following the manufacturer’s protocol. A total of 120,429,967 pairs (125 bp) were sequenced on an Illumina HiSeq1500. An initial read quality check in FastQC (http://www.bioinformatics.babraham.ac.uk/projects/fastqc/) identified a low-quality stretch of the first 8 bp in each read, which was clipped with Trimmomatic v0.35 [84] (added options: HEADCROP:8]. Clipped reads were subsequently filtered using the BioLite v0.4.0 filtering tool [85] [added options: -q 28 -t 33 -a -b]. All reads with an average Phred Quality Score below 28 and/or reads with vector contamination were removed entirely without trimming. Quality filtering reduced the dataset to 103,388,888 high-quality reads (2 × 117 bp; total 24.2 Gb, equaling approximately 277x genome coverage).

#### Moleculo long-read sequencing

Moleculo reads were prepared using the TruSeq Synthetic Long-Read DNA Library Prep kit following the manufacturer’s protocol (Illumina, San Diego, USA). A total of 500-ng high-molecular-weight genomic DNA was used as input for the library preparation. Two lanes of the barcoded library were sequenced on an Illumina HiSeq1500 run and assembled using Illumina’s cloud-based service (BaseSpace Sequence Hub). A total of 83,688 Moleculo reads >500 bp were generated with an N50 of 5.4 kb, a peak at 8 kb, and a total size of 320 Mb. Trimming of low-quality and vector regions was performed with Geneious R8 [86] [added options: error probability limit 0.01; maximum low-quality bases 80; maximum ambiguities 4] and resulted in 79,974 high-quality Moleculo reads >500 bp (totaling 313 Mb). Moleculo reads assembly in Geneious R8 [added options: minimum overlap of 400 bp; 100% identical overlaps; no gaps allowed] resulted in 49,793 assembled sequences (contigs and singlet) with an N50 of 7.5 kb (total 258 Mb equaling approximately 2.9x genome coverage).

#### dipSPAdes hybrid assembly

A mixed read type assembly was performed with the SPAdes 3.5.0 package [87,88]. Filtered paired-end reads were error corrected within the assembly pipeline, which consists of (1) error correction, (2) SPAdes haplocontig assembly, and (3) dipSPAdes haplocontig merging. The assembled Long Artificial Reads were input as “-trusted contigs” [other added options:—cov-cutoff 10—careful -k 39,49,59,69,79,89,99,109. dipSPAdes merging resulted in a total of 777 contigs >500 bp].

#### Contamination screening

dipSPAdes haplocontigs were screened for bacterial contaminations by TBLASTN searches (evalue 1e−10) using proteins from the *Candidatus Midichloria mitochondrii* (order Rickettsiales) genome, the bacterial species most closely related to the previously identified *T*. *adhaerens* endosymbiont [89]. In a second TBLASTN search, we used plasmid-encoded proteins from all Rickettsiales genomes at NCBI (May 2016) to determine putative plasmid-associated contigs. All candidate bacterial chromosome and plasmid contigs (*n* = 19) were re-BLASTed (BLASTN and TBLASTX) against complete Rickettsiales genomes to confirm the bacterial origin and were subsequently removed from the *H*. *hongkongensis* nuclear genome assembly. The mitochondrial chromosome was further identified by BLASTN searches (evalue 1e−20) using the haplotype H15 mitochondrial genome [90] (Genbank accession NC_015309.1) and also removed from the nuclear genome contigs. The circular *H*. *hongkongensis* mitochondrial genome has a size of 36,537 bp and shares a 1-bp exon in the cox1 gene with other placozoans [91]. It shares all genes and has the identical gene order as the two already published placozoan mitogenomes of clade V (haplotypes H4 and H15; [61,90]). The complete and annotated *H*. *hongkongensis* mitochondrial genome was deposited in the genome repository (https://bitbucket.org/molpalmuc/hoilungia-genome/src/master/mitochondrial_genome/). Automatic annotation of the mitochondrial genome was performed with the MFannot web server [92] and corrected based on the other available placozoan mitochondrial genomes.

#### Supercontig generation

After removing contaminants, we assembled supercontigs. In the first place, 50 bp were clipped off from both ends of all dipSPAdes consensus contigs, as the coverage toward the ends of contigs drops, and errors might accumulate. After clipping, contigs <500 bp were removed. Remaining contigs were assembled in Geneious R8. To identify correct overlaps, *ab initio* gene models were generated for the contigs before assembly with AUGUSTUS 3.0.3 [93]. AUGUSTUS was trained online using the WebAUGUSTUS service (http://bioinf.uni-greifswald.de/webaugustus) using the clipped genomic contigs and a reduced set of Trinity transcripts (see section "Transcriptome assembly"). This set only included “c0_gi_i1” components of all transcripts and consisted of 33,708 transcripts. After the training, AUGUSTUS was run with the resulting species parameter output [added options: species = placo_h13, strand = both, genemodel = atleastone, codingseq = on, protein = on, cds = on, sample = 100, keep_viterbi = true, alternatives-from-sampling = true, minexonintronprob = 0.2, minmeanexonintronprob = 0.5, maxtracks = 10, GFF3 = on, exonnames = on].

Settings used in the Geneious supercontig assembly were 5-kb minimum overlap, 2% maximum mismatch per contig, 2% maximum gaps per contig, 2,000-bp maximum single gap size (to account for larger indels), and 40-bp word length. Overlapping contigs were checked in Geneious for identical exons/intron structure of predicted AUGUSTUS gene models in the overlap. In case of <100% overlap sequence identity, one or both contigs were trimmed manually to keep a 100% identical overlap. Consensus supercontigs were then called in Geneious.

Even after the dipSPAdes merging step and the Geneious assembly, some overlapping haplocontigs were identified by all against all BLASTN searches of the supercontigs. Merging of these haplocontigs was performed with the second round of Geneious supercontig assembly with less stringent settings: 5-kb minimum overlap, 25% maximum mismatch per read, 15% maximum gaps per read, 2,000-bp maximum single gap size, and 24-bp word length. Overlapping contigs were again checked for identical AUGUSTUS gene models. In the case of missing annotation on both sequences, BLASTN searches of both haplocontigs were performed against all supercontigs. Haplocontigs were merged if both sequences hit itself or the overlapping haplocontig only. Trimming of overlaps was carried out as mentioned above. Supercontig consensus calling was done in Geneious with default settings. Overlapping contigs with insertions in one contig of up to 2 kb were merged based on Moleculo read support. For this Moleculo, reads were mapped to the supercontigs in Geneious in “low stringency” mode.

A third Geneious assembly was performed to remove internal allelic redundant contigs, i.e., haplocontigs with full overlap to a supercontig. Low stringency settings for this final Geneious assembly were 0.5-kb minimum overlap, 25% maximum mismatch per read, 15% maximum gaps per read, 2,000-bp maximum single gap size, and 24-bp word length. Both the entirely overlapping (internal redundant) haplocontig, as well as the partially overlapping contig, were used for BLASTN searches against all supercontigs to confirm matches of the full-length overlap in only two highly confident (1e−100) BLAST hits. In addition, internal allelic contigs were confirmed by identical AUGUSTUS models on both alleles. Confirmed internal allelic (redundant) contigs were then removed.

This procedure ended in a genomic assembly of 669 gap-free supercontigs with an N50 of 407.8 kb and a total of 87,194,036 bp. These contigs are hereafter termed "reference contigs." Additional scaffolding was not performed, as Moleculo reads bridged most complex regions, and no additional reads were available for further scaffolding. For *H*. *hongkongensis* assembly and annotation statistics and a comparison to the *T*. *adhaerens*, see S1 Table in the main text. We created versions of the reference contigs with repeats hardmasked and softmasked in RepeatMasker 4.0.6 [94] [added options: -s -norna -a -inv -lcambig -source -html -gff -e hmmer & -small for softmasking] using the "*T*. *adhaerens*" reference of the RepeatMasker RepBase database.

### Transcriptome sequencing and assembly

#### Library preparation and sequencing

RNA was extracted from the RNA strain in 2 batches of 100 clonal individuals each, using standard phenol/chloroform extractions. RNA was shipped to the New York Genome Center (New York, NY, USA) for RNA quality check, library preparation, and sequencing. Strand-specific libraries were prepared with 500 ng total RNA using the TruSeq stranded mRNA V2 kit (Illumina, San Diego, USA). The nominal library insert size was 300 bp. A total of 61,313,870 strand-specific 125-bp RNA pairs (13.1 Gb) were sequenced on an Illumina HiSeq2500.

#### Transcriptome assembly

Prior to Trinity assembly, reads were quality checked in FastQC and filtered with BioLite 0.4.0 [added options: -q 25 -t 33 -a -b], keeping all reads with an average Phred Quality Score >25. This reduced the number to 57,237,523 high-quality read pairs. Reads were assembled with Trinity v2.0.6 [95,96] [added options:—seqType fq—SS_lib_type RF—normalize_reads—trimmomatic—max_memory 50G]. A total of 124,155 transcripts were assembled with an N50 of 2,550 bp and an average length of 1,506 bp.

### Genome annotation

#### Genome-based transcript generation

Filtered (see section “Transcriptome assembly”) strand-specific paired RNA reads were mapped to the hardmasked reference contigs with Tophat2 v2.1.0 [97] [added options:—library-type fr-firststrand]. The Tophat2 output bam file was used to run StringTie v1.2.2 [98] with default settings on the hardmasked reference contigs. Finally, StringTie transcripts and predicted protein and encoded protein sequences were created with TransDecoder v2.1 [96] and default settings.

#### *Ab initio* gene prediction

The softmasked reference contigs were run in the BRAKER1 v1.9 [99] pipeline with default settings using the Tophat2 bam file of mapped RNA-Seq reads as guidance. BRAKER1 predicted 12,010 genes and 12,575 transcripts (S1 Table).

#### Identification of unexpressed *ab initio* gene models

To calculate the amount of unexpressed *ab initio* BRAKER1-predicted proteins, we identified their overlap with StringTie and Trinity transcripts using BEDtools intersect [added options: -s -v -f 1E-4 -r]. Gene model IDs, extracted from the resulting table, were used to extract expressed (models with overlapping/coincident RNA-Seq-based transcripts) and nonexpressed gene models from the BRAKER1 annotation GFF file. Of the 12,010 BRAKER1 genes, only 422 (3.5%) were not expressed.

#### Functional annotation

The *H*. *hongkongensis* and the *T*. *adhaeren*s *ab initio* proteomes were annotated with Blast2GO [100]. Local BLASTP searches [evalue 1e−3] were performed against metazoan UniProt proteins (http://www.uniprot.org/, [101]), followed by mapping and annotation. To identify Pfam and ProDom domains, we ran InterProScan v65.0 [102] [added options: -f xml -goterms -iprlookup -appl Pfam,ProDom]. Identified domains were merged with annotations in Blast2GO, and final annotations were extracted (S1 & S3 Data).

We separately annotated the StringTie transcripts by local BLASTX searches [103] of the transcripts against (1) *T*. *adhaerens* reference proteins from NCBI, (2) UniProt proteins, and (3) *H*. *hongkongensis-*predicted BRAKER1 proteins [added options in all cases: -evalue 1e-10 -max_target_seqs 2 -outfmt 6]. For BLAST searches, the standalone BLAST+ suite v2.6 [104] was used. To identify domains in the *H*. *hongkongensis* proteome, we performed an HMMscan on the StringTie transcripts using hidden Markov models (HMMs) of Pfam-A release v30.0 [105] with HMMER v3.1b2 [106,107]. The resulting table (S2 Data) was used to generate a GFF3 annotation file of the domains based on the StringTie transcripts with a custom Python script (pfam2gff.py). A combined BLAST and Pfam annotation table was created using a custom Python script (collectannotationinfo.py). In addition to StringTie transcripts annotation information, this second table also includes exon counts, gene position, and gene expression (fragments per kilobase million [FPKM] and transcripts per kilobase million [TPM]) information.

tRNAs were predicted with tRNAscan-SE on the reference contigs with default settings and stored in an annotation GFF3 format.

### Genome coverage

A "lavalamp" kmer/GC plot was generated (S2 Fig) to yield a high-resolution plot of read counts per %GC and 31 bp kmer coverage using the Jellyfish kmer counter and a set of custom Python scripts (kmersorter.py and fastqdumps2histo.py; for details on the procedure, see https://github.com/wrf/lavaLampPlot). In contrast to the conceptually similar approach Blobtools [108], we used raw reads instead of contigs to yield a high-resolution plot of read counts per %GC and 31 mer coverage. The plot identified two read clouds with high counts at a kmer coverage of 80–140x (heterozygous “read cloud”) and 160–260x (homozygous “read cloud”), respectively. Additional “read clouds” at 270–320x and 380–410x coverage mark repetitive sequence stretches. Another “read cloud” was found at a low coverage of 20–50x. Reads within this cloud and their pairs were extracted with kmersorter.py [added options: -s 0.16 -b 50 -w 0.40 -T -k 31] and fastqdumps2histo.py. Bowtie2 v2.2.5 [109] [added options: -q—no-sq] was used to map the 580,092 extracted reads to the 19 previously identified bacterial contigs (see section “Contamination screening”). More than 86% of these reads mapped to the bacterial contigs, confirming the bacterial origin of the reads within the low-coverage “read cloud.” Read counts identified a relatively high abundance of bacterial cells, and the GC content was similar to the host genome.

To estimate the per-base genome coverage, paired-end reads were mapped to the softmasked reference assembly with Bowtie2 v2.2.5 [added options: -q—no-unal—no-sq) and sorted with SAMtools v1.3.1 [110]. The bam file was used to create a bedgraph file in BEDtools v2.25.0 [111] by invoking the genomecov operation [added options: -ibam stdin -bga]. A custom Python script (bedgraph2histo.py) [added options: -m 2000] was used to create a coverage histogram table. Since 81.4% of the genome falls within the second peak (165–332x coverage with a maximum at 248x), most of the genome was merged in the reference assembly (S3 Fig).

### Genome completeness

#### Read and transcript mapping

To estimate the completeness of the reference assembly, we first mapped paired-end reads and Moleculo reads back to the reference genome. For paired-end read mapping, see section “Genome coverage.” BWA v0.7.12 [112] was used to map the Moleculo reads. Two successive rounds of mapping were performed with BWA mem. The first with stringent settings for long reads [added options: -k 200 -w 16000 -x intractg]. The output was filtered with the SAMtools v1.3.1 view script to receive mapped and unmapped reads. The 12,271 unmapped reads were mapped again using lower stringency settings to account for lower sequence identity in intergenic regions [added options: -w 16000 -x intractg]. More than 93% of the Moleculo reads and 84% of paired-end reads mapped back to reference contigs, indicating a highly complete reference genome assembly and a low misassembly rate. For RNA-Seq read mapping with Tophat2, see section “Genome-based transcript generation.”

Trinity transcripts and transdecoder-predicted protein-coding sequences were mapped to the hardmasked genome with GMAP v2015-07-23 [113] [added options: -f 3 -B 5 -n 1—cross-species]. All DNA, RNA, and transcript-mapping stats are summarized in S3 Table.

#### BUSCO v3 gene set screening

To further evaluate genome completeness, we screened for a set of single-copy proteins conserved in all animals, the BUSCO gene set. BUSCO v3.02 [114] [added options: -l metazoa_odb9] was run separately on the *ab initio* (BRAKER1) proteins and the StringTie and Trinity transcriptome and transdecoder proteins, respectively.

### Synteny and gene collinearity

To identify collinearity between the two placozoan species, all *H*. *hongkongensis* contigs >100 kb were aligned to the longest 10 *T*. *adhaerens* scaffolds (accounting for 70.3 Mb or 66.5% of the genome assembly; including 5.7-Mb gaps) with default settings. For generating the alignments, LASTZ v1.02.00 [115] (implemented as a plugin in Geneious) was used. Of the 222 *H*. *hongkongensis* contigs >100 kb, a total of 144 (accounting for 60.6 Mb or 69.4% of the genome assembly) aligned to the 10 longest *T*. *adhaerens* scaffolds. Aligned *H*. *hongkongensis* contigs were extracted from the assembly, sorted, and occasionally reverse complemented to be oriented according to the *T*. *adhaerens* scaffolds. Gene annotations (GFF) of contigs as well as protein sequences were extracted for the target scaffolds/contigs sets of both species. A MCScanX run [116] [added option: -a] was performed for each target set, using the extracted *T*. *adhaerens* and *H*. *hongkongensis* GFFs together with the reciprocal best 5 BLASTP hits [added options: -evalue 1e-10 -max_target_seqs 5 -outfmt 6] between and among proteins of both placozoans. Dual synteny line plots of the resulting collinearity files were visualized in VGSC v1.1 [117] [added options: -tp DualSynteny] and combined to Fig 2A. In addition, bar plots were generated for the 10 *T*. *adhaerens* scaffolds and the matching 144 *H*. *hongkongensis* contigs in VGSC [added option: -tp Bar]. Bar plots were mapped onto the DualSyntheny plots to show collinearity within each set and macrosynteny between both genomes. The percentage of collinearity between the *T*. *adhaerens* scaffolds and *H*. *hongkongensis* contigs was calculated in MCScanX, and results for the 10 scaffolds are given in S5 Table. The mean collinearity was calculated as the sum of the individual collinearities for the 10 *T*. *adhaerens* scaffolds multiplied by a size-correction faction for each scaffold (i.e., percent coverage of the evaluated 70.4 Mb of the *T*. *adhaerens* genome).

Syntenic block sizes and the number of blocks were calculated using the custom Python script microsynteny.py (described in [118]) with skipping no more than 1 gene [added option: -s 1] and otherwise default options.

### SNPs

#### Genomic SNPs

SNPs were identified with two alternative tools, FreeBayes v0.9.21 [119] and GATK v3.5 [120,121]. For both analyses, the bam file of Bowtie2 mapped reads (see section “Genome coverage”) was used as input.

FreeBayes was run in parallel mode, and the resulting vcf file was filtered with VCFfilter [added options: -f "QUAL > 20"]. For the GATK analysis, the GATK best-practice guidelines for variant discovery in DNAseq were followed (https://software.broadinstitute.org/gatk/best-practices/). Initially, an index of the reference contigs was generated with SAMtools and a dictionary file with the Picard Tools v 2.3.0 CreateSequenceDictionary script (http://broadinstitute.github.io/picard). Read groups were then defined, reads sorted, duplicates marked, and an index created with the Picard Tools scripts AddOrReplaceReadGroups [added options: SO = coordinate) and MarkDuplicates [added options: CREATE_INDEX = true, VALIDATION_STRINGENCY = SILENT, M]. Processes files were used for the successive GATK variant calling using a set of scripts. Base frequencies were recalibrated with BaseRecalibrator [added options: -nct 8, -knownSites], using the FreeBayes vcf (see section "Genomic SNPs") as recalibration input. A second pass was run using the produced recalibration table to analyze covariation remaining after recalibration. As recalibration improved read quality scores, the recalibration was applied to the sequence data with PrintReads [added options: -nct 8, -I, -BQSR]. Variants were then called using the recalibrated reads with HaplotypeCaller [added options: -nct 8,—genotyping_mode DISCOVERY, -stand_call_conf 10 -stand_emit_conf 30]. SNPs were extracted from the call set with SelectVariants [added options: -selectType SNP]. Highly stringent SNP filtering was performed with VariantFiltration [added options:—filterExpression "QD < 2.0 || FS > 60.0 || MQ < 40.0 || MQRankSum < -12.5 || ReadPosRankSum < -8.0"]. Indels were extracted from the variant call set with SelectVariants [added options: -selectType INDEL] and filtered with VariantFiltration [added option:—filterExpression "QD < 2.0 || FS > 200.0 || ReadPosRankSum < -20.0"]. This procedure identified 1,397,488 high-confidence genomic SNPs in the *H*. *hongkongensis* DNA, equaling roughly 16 SNPs per 1 kb, or a heterozygosity of 1.6%.

To identify SNP in the exonic, intronic, and intergenic fraction of the genome, the FreeBayes vcf (see section "Genomic SNPs") was input in a custom Python script (vcfstats.py) together with the StringTie annotation gtf and the StringTie transdecoder annotation GFF file (see section "Genome-based transcript generation" for details). A plot of the SNP numbers against the coverage identified the heterozygous and homozygous peaks with differences in SNPs between the genomic fractions (S4 Fig). The exonic portion showed almost no SNPs within the heterozygous and the highest number in the homozygous peak, whereas the intergenic fraction had a more substantial amount of SNP in the heterozygous and a reduced amount in the homozygous peak. The intronic portion is an intermediate between the two. This indicates that (a) most of the genic (exonic and intronic) regions have been successfully merged in the assembly process, resulting in an almost completely merged reference assembly, and (b) the proportion of unmerged haplocontigs is essentially higher in the intergenic fraction. This confirms an expected higher sequence divergence between the two genomic haplotypes in intergenic regions.

#### SNPs in RNA-Seq data

To call RNA-Seq variants, the GATK best-practice guidelines for variant calling on RNA-Seq was followed [121,122]. The Tophat2 RNA-Seq mapping bam file (see section "Genome-based transcript generation") was used. The index and dictionary files were generated as for DNA SNPs (see section "Genomic SNPs"). Read groups were defined, reads sorted, duplicates marked, and an index created with the Picard Tools, as mentioned. Process files were used for the successive GATK variant calling, using a set of scripts. To split reads into exon segments, hard-clip any sequences overhanging into the intronic regions, and to reassign mapping qualities, the SplitNCigarReads script was applied [added options: -rf ReassignOneMappingQuality -RMQF 255 -RMQT 60 –U ALLOW_N_CIGAR_READS]. Base recalibration (one round) and read printing were performed as for DNA. Variant calling of recalibrated reads was done with HaplotypeCaller [added options: -dontUseSoftClippedBases -stand_call_conf 10.0 -stand_emit_conf 30.0] and stringent filtering with VariantFiltration [added options: -filterName FS -filter "FS > 30.0" -filterName QD -filter "QD < 2.0"]. This procedure identified 302,430 high-confidence SNPs, or 1 unique SNP per 1-kb CDS between the *H*. *hongkongensis* strains, indicating a low polymorphism rate between populations from different sampling sites.

#### Comparison of genomic and transcriptomic SNPs

SNP numbers and sites were compared between the two *Hoilungia* strains. First, all identified DNA and RNA SNPs within predicted BRAKER1 exons were extracted separately with BEDtools intersect [added options: -a -b -wa]. Second, unique DNA and RNA SNPs were extracted with BEDtools intersect [added options: -a -b -v -f 1.0 -wa]. This procedure identified a total of 138,302 (45.7% of all) RNA SNPs in exons, 21,963 (15.7%) of which are unique to strain M153E-2. This is the equivalent of 1 unique SNP per kilobase CDS. In contrast, a total of 202.901 (14.5% of all) DNA SNPs were identified in exons in the DNA strain, with 86,278 (6.2%) unique exonic SNPs or 4 SNPs per kilobase CDS. Combined SNP counts indicate shallow differences between the two strains, with only 0.5% unique SNPs in the CDS. The number of intronic regions is expected to be higher, but as no genomic data is available from M153E-2, this cannot be tested. All SNPs counts are summarized in S2 Table.

### Identification of allele sharing and reproductive isolation

To identify allele sharing or reproductive isolation, 3 genes encoding for ribosomal proteins were amplified via PCR, using degenerate primers designed based on the *T*. *adhaerens* genomic sequence, as well as a previously sequenced EST library of lineage H4 [19]. Primer sequences to amplify gDNA (including intronic sequence) for the ribosomal proteins L9 and L32, as well as ribosomal protein P1, were as follows:

rpl9-Fw:TTGCAGCCATGAAGACYATAYTGTC;
rpl9-Rv:GAATCWGCATTMCRAAATGTACAC;
rpl32-Fw:AAATGGTCACTCCAGTAAATAAGC;
rpl32-Rv:AGTTMATCCATAAAATACAATTACAGT;
rpp1-Fw:AGGTGTATCGTTCTCTTGTAGCTAAG;
rpp1-Rv:ATATCMTCGTCAGATTCTTCWTCTTC.

PCRs were run with an initial denaturation of 3 min at 94 °C; followed by 40 cycles of 30 s of denaturation at 94 °C, 30 s of annealing at 60 °C, and 1.5 min of elongation at 72 °C; and finished with a final elongation for 3 min at 72 °C. The BIOTAQ system was used (Bioline, London). A list of samples used for amplification is provided as S6 Table. Sequencing was performed by Macrogen (South Korea). Alleles were identified as double peaks in standard sequencing in the case of heterozygous alleles. The phasing of SNPs was inferred from homozygous sequences as well as the sequence of allelic variants in closely related haplotypes, for which phasing information was available because of the long Sanger reads.

To check for reproductive isolation and to identify conspecific isolates, haplowebs [123] were generated for each marker as well as a CM [54] for combined markers using the online tool HaplowebMaker (https://eeg-ebe.github.io/HaplowebMaker/; Spöri & Flot, in prep.). The resulting conspecificity scores were plotted in R using the heatmap3 package [124], sorted according to a UPGMA tree (JC69 model) of the three concatenated genomic sequences (with indels removed). If present, both alleles of an isolate were merged, and the consensus sequence was used to generate the tree.

### Intra- and interspecific placozoan distances

#### Interspecific distance calculations

Protein sequences of all 6,644 MCL-predicted one-to-one orthologs for *H*. *hongkongensis* and *T*. *adhaerens* were aligned with MAFFT [added options:—einsi], and genetic identities were called in ClustalO v1.2.0 [125] [added options:—percent-id—full—output-order = input-order—distmat-out]. Distance percentages were calculated based on resulting identities. The nucleotide CDSs were back-aligned based on the untrimmed protein alignment, using a custom Python script (regapper.py). Because of the highly diverged protein sequences, 90 orthologs could not be unambiguously re-gapped and were removed from the set.

#### Intraspecific distance calculations

To identify all loci with both full-length alleles available, we extracted all reference gene sequences (CDS and introns) plus 1-kb sequences upstream and downstream based on the BRAKER1 annotation GFF file. Only the longest gene model was used for each gene. Haplocontigs generated by SPAdes (the first step in the dipSPAdes assembly pipeline) were mapped against the extracted reference gene sequences with BWA mem [added options: -k100 -W40 -r10 -A1 -B1 -O1 -E1 -L0]. Unmatched regions of the haplocontigs were hard clipped with Bamutils removeclipping of the NGSUtils v0.5.7 [126] with default settings. This also trimmed the overhanging haplocontigs to the reference sequence length. After a size-filtering of mapped contigs with Bamutils filter [added options: -minlen 1000], the bam file was sorted with SAMtools view. All alignments were loaded into Geneious R8 and filtered to keep only loci with (1) 100% reference coverage, (2) precisely 2 mapped haplocontigs, and (3) both haplocontigs spanning the BRAKER1 gene model in the reference. This resulted in 5,401 loci for which the reference and both allele sequences were extracted and gaps removed. Subsequently, RNA-Seq data were mapped to the three sequences (reference sequence plus 2 haplocontigs) for each of the 5,401 loci with Tophat2. The BRAKER1 pipeline was then run with the generated RNA-mapping bam file with changes in some BRAKER1 scripts: (1) “—min_contig = 100” was added to the GeneMark-ET script (line 616) to perform training on contigs with at least 1 kb (instead of 50 kb), and (2) “—alternatives-from-evidence = $alternatives_from_evidence” was replaced by “—genemodel = exactlyone” in the BRAKER1 script to predict only one gene for each allelic contig. CDSs from the BRAKER1 predictions were extracted and assembled in Geneious, allowing for 20% sequence difference, 20% gaps, 500-bp gap size, and multiple mapping. Loci with more or fewer than 3 sequences were excluded from further analyses. This resulted in 4,452 loci with full-length gene models (termed “full-length loci set”) with precisely 3 *H*. *hongkongensis* sequences each (reference, allele A, and allele B).

The full-length loci set was then filtered based on the one-to-one ortholog IDs calculated in MCL. This procedure finally resulted in 2,870 high-confident and full-length one-to-one orthologs between *H*. *hongkongensis* and *T*. *adhaerens* in addition to both full-length allelic variants in *H*. *hongkongensis*, which were used for alignments and distance calculations as described in section "Interspecific distance calculations."

### dN/dS ratios and codon saturation

dN/dS ratios—as well as fractions of unchanged codons, synonymous, and nonsynonymous sites—were calculated based on a custom Python script (alignmentdnds.py) using regapped CDS alignments and untrimmed protein alignments (S6 Fig). Codons with any ambiguous bases and gapped sites were ignored.

### Gene clustering with *T*. *adhaerens*

Clustering into homologs and co-orthologs was performed with a custom python script (makehomologs.py) [added options:-s 1 -p 234 -H 200]. The script calls the MCL v12-068 algorithm [127], which uses the output of a local all-versus-all BLASTP search [added options: -evalue 1e-3 -outfmt 6] of all *H*. *hongkongensis* and *T*. *adhaerens* proteins.

### GO term enrichment analyses

To identify enriched GO terms in non-BLAST hits as well as in four co-ortholog categories (one-to-many, many-to-one, many-to-many, and many-to-zero), an enrichment analysis was performed for the three main GO categories (Biological Process, Cellular Component, and Molecular Function) using topGO [128]. Only enriched GO terms with a *p*-value <0.05 were kept, based on the classic Fisher test.

Ortholog categories (see also [40]) are defined as (1) one-to-one: Only one ortholog is found in each species; (2) one-to-many: One ortholog in this species, but many co-orthologs in the other species. The gene was duplicated in the other species from the ancestral copy after speciation; (3) many-to-one: More than one co-ortholog in this species but only one in the other species. The gene was duplicated in this species from the ancestral copy after speciation; (4) many-to-many: More than one co-ortholog in this and the other species. At least two gene duplications could be found from an ancestral gene in the common ancestor of both species—one duplication in this species, and a second one in the other species; (5) many-to-zero: Many co-orthologs in this species but none in the other. In this case, the gene was duplicated from an ancestral copy in this species after speciation and likely lost in the other species.

### Cross-phylum distance comparison at various taxonomic levels

To estimate molecular differences between *H*. *hongkongensis* and *T*. *adhaerens* and to bring these into a taxonomic context, we measured genetic distance using an extended data matrix of 212 nuclear proteins set up by Cannon and colleagues [55]. This data matrix was chosen as it includes a comparable number of sites for a diverse taxonomic range and is, therefore, also suitable for phylogenetic analyses. In addition, genetic distances were measured for 5 standard barcoding (“selected”) markers—namely, nuclear ribosomal subunits 18S (S9 Fig) and 28S (S10 Fig), mitochondrial large ribosomal subunit 16S (S11 Fig), and the mitochondrial proteins cytochrome c oxidase subunit 1 (CO1) (S12 Fig) and NADH dehydrogenase subunit 1 (ND1) (S13 Fig). An overview of means for all distances of all six marker sets is provided as S14 Fig. The incorporation of datasets from four individual categories (nuclear protein versus nuclear rDNA versus mitochondrial protein versus mitochondrial rDNA) enabled the comparison among markers with different substitution rates.

#### Ortholog identification and alignment of nuclear proteins

Orthologs of the 212 proteins were identified for *H*. *hongkongensis*, *T*. *adhaerens*, and a set of selected sponges, cnidarians, and ctenophores in a two-step process. First, HaMStR was used to identify orthologs. Transcriptomes were either downloaded from respective sources, or, if no transcriptome was available, an assembly was generated with Trinity v2.0.6 [added options:—normalize_reads—trimmomatic]. All used transcriptomes were translated using a custom Python script (prottrans.py), keeping only proteins with at least 50 amino acids [added options: -r -m -n -a 50]. HMMs were built for all genes based on the final Cannon and colleagues protein alignments, with HMMER to perform ortholog searches. Using the sequences included in their alignments, reference BLAST datasets were created for the two outgroups (*Monosiga brevicollis*, *Salpingoeca rosetta*), all nonbilaterians (*T*. *adhaerens*, *Amphimedon queenslandica*, *Leucosolenia complicata*, *Aphrocallistes vastus*, *Oscarella carmela*, *Craspedacusta sowerby*, *Nematostella vectensis*, *Stomolophus meleagris*, *Euplokamis dunlapae*, *Mnemiopsis leidyi*, *Pleurobrachia bachei*), plus *Drosophila melanogaster* and *Homo sapiens*. The first HaMStR run was performed on the translated unigenes of a limited broad-range taxon set, which included representatives from all nonbilaterian phyla and all classes within these, when available. In this first run, the mentioned 15 reference taxa mentioned were used [added options: -eval_hmmer = 1e-10 -eval_BLAST = 1e-10 -representative -append -strict]. HaMStR outputs were transformed to fasta format, and redundant orthologs of the 15 HaMStR runs for each proteome were filtered with a custom Python script (commonseq.py) [added options: -t p]. Sequences of individual ortholog groups for all taxa were combined to separate fasta files, which were aligned with the respective untrimmed alignment (kindly provided by Johanna Taylor Cannon) using MAFFT v7.273 [81] [added options: -linsi—amino—leavegappyregion]. Trimmed sequences from the Cannon and colleagues 212-gene set were aligned to the first alignment again with MAFFT and the same options. This procedure enabled accurate alignment of the trimmed sequences with the newly added sequences. The second alignment was trimmed according to the included trimmed sequences and used to create a second set of HMMs and BLAST reference taxa for another HaMStR run on the remaining proteomes. In this run, we used *M*. *brevicollis*, *S*. *rosetta*, *D*. *melanogaster*, and *H*. *sapiens* as core reference taxa, plus an individually selected set of reference taxa for the four nonbilaterian phyla: (a) each one taxon of the Anthozoa, Hydrozoa, Scyphozoa, and Cubozoa for Cnidaria; (b) each one reference taxon of the Calcarea, Hextactinellida, and Homoscloromorpha, as well as two of the Demospongiae for Porifera; (c) *P*. *bachei* and *M*. *leiydi* for Ctenophora; and (d) *T*. *adhaerens* for Placozoa. Final alignments for orthologs were generated as stated before.

We carefully curated every single protein by generating single-gene trees to identify contaminations and paralogs in the original Cannon and colleagues 212-protein dataset as well as in the newly added data. Filtering of paralogs was performed in PhyloTreePruner [129] based on trees generated with FastTree v2.1.5 [130] using default settings.

Based on this approach, we identified a high rate of contamination in several parasitic as well as free-living cnidarians and in one Ctenophore. The transcriptomes/proteomes of the following taxa (Genbank accessions in parentheses) were excluded because of a high load of contaminations and are therefore not listed in S7 Table: *Myxobolus cerebralis* (SRP045736), *Myxobolus pendula* (SRP063943), *Kudoa iwatai* (SRP042325), *Thelohanellus kitauei* (SRP020474), *Polypodium hydriforme* (SRP042947), *Platygyra carnosus* (SRP010342), *Podocoryne carnea* (SRP041583), *Coelastrea aspera* (ERP105121), *Acropora formosa* (SRP103173), *Acropora cerealis* (SRP103173), *Heliopora coerulea* (SRP115860), *Balanophyla europaea* (SRP075606), and *Pukia falcata* (SRP114767).

After pruning, alignments were inspected manually, and misaligned sequence ends were trimmed to the next unambiguously aligned position with respect to the next closest related taxa. This two-stage HaMStR approach using a broad phylogenetic range of reference taxa in the first and multiple selected taxa in the second run resulted in a higher yield of orthologs compared to a single run with a single and distantly related taxon (e.g., *D*. *melanogaster*) alone.

After the second round of HaMStR ortholog identification and alignment processing, the final protein alignments were used to screen an extended set of Cnidaria, Porifera, and Ctenophora, in addition to all available chordate taxa from the classes Actinopteri, Aves, and Mammalia with a sequenced genome. Inclusion of taxa from the other two chordate subphyla (Tunicata and Cephalochordata) was omitted, since genomic information for these groups is scarce, and/or only low-quality sequence data were available (e.g., annotations of the two available Tunicate genomes were highly incorrect for a majority of the 212 genes).

For this, the second step of ortholog identification, a custom script (add_taxa_to_align.py) [added option:—ev-allowance 1e35] was used to identify and automatically align orthologs based on HMM profiles of the trimmed alignments resulting from the HaMStR searches. After screening of >500 taxa (of Cnidaria, Porifera, Ctenophora, and Chordata), each alignment was processed manually, as described for the HaMStR searches. From the final concatenated alignment, we removed all taxa with fewer than 30% of sites of the full matrix (37,838 amino acid sites). The final set of 378 taxa used for distance calculations is given in S7 Table. We refer to the final alignment as dataset 1 (see S9 Table for an overview of the 2 datasets).

#### Ortholog identification and alignment of selected barcoding markers

Mitochondrial markers were extracted from public mitochondrial genomes if available (S11 Table). To retrieve mitochondrial genes from taxa without published mitochondrial genomes, we performed BLASTN/TBLASTX (evalue 1E-5) searches against available transcriptomes (S7 Table). Nuclear rDNA sequences were identified by BLASTN searches against transcriptomes, using the rDNA sequence of the next closest related taxa for which sequence information was available. For all included Porifera, Cnidaria, and Ctenophora taxa, we could isolate full-length 18S and 28S sequences from transcriptomic/genomic data and, in most cases, even the full-length rDNA cascade (including ITS1/2 and 5.8S). We used the placozoan rDNA accessions AY652583.1, AY652578.1, AY652585.1, AY652580.1, AY652587.1, AY652581.1.

Multiple sequence alignments were generated with MAFFT using the LINSI algorithm for protein sequences (CO1, ND1) and the GINSI algorithm for ribosomal genes (16S, 18S, 28S) with otherwise default settings. Individual alignments were created for each class within Porifera and Cnidaria to reduce unambiguously aligned sites. For the Placozoa and the Ctenophora, we used all sequences to generate a single alignment for each marker.

#### Distance calculations

Mean group pairwise genetic distances were calculated in MEGA7 [131] [settings: model/method = p-distance; gaps/missing = pairwise]. Groups were assigned to all taxa, and between-group mean distances were calculated for orders within classes, families within orders, and genera within families for the nonbilaterian phyla Porifera, Cnidaria, and Ctenophora. The nuclear protein distance in placozoans was haponly calculated for *T*. *adhaerens* and *H*. *hongkongensis*, since no other genomes are available.

To calculate genetic distances of selected single gene markers within the Placozoa, we included two additional undescribed placozoan haplotypes (H4 and H8). These two taxa were added for a better representation of genetic distances within the entire phylum. According to the established placozoan 16S molecular phylogeny [5], *H*. *hongkongensis* and Placozoa sp. H4 represent closely related taxa within the placozoan “subgroup A2,” Placozoa sp. H8 represents “subgroup A1,” and *T*. *adhaerens* represents “group B.”

### Phylogenomics

To assess the effect of adding a second placozoan species on the placement of the Placozoa in the animal tree of life and to estimate branch lengths to the two placozoan species, dataset 1 was further condensed to generate a highly complete protein matrix (dataset 2). This set had only 10.8% missing characters in 58 taxa, including 32 nonbilaterians and 2 outgroups with an almost complete gene set (194 genes, see also gene density matrix in S21 Fig).

It has been demonstrated that the CAT model (specifically CAT-GTR) implemented in PhyloBayes [132] fits phylogenomic amino acid supermatrices containing nonbilaterians best [73,133], and obviously, only best-fitting evolutionary models should be used in probabilistic phylogenetic analyses to reduce systematic errors [133]. However, the computational burden of reaching convergence of analyses using the CAT-GTR model can be prohibitive. It is also well known that phylogenomic datasets frequently suffer from compositional heterogeneity that might negatively influence phylogeny estimation [134–136]. Compositional heterogeneities can be reduced by the so-called Dayhoff recoding [65,137,138], which combines amino acids with similar physicochemical properties into one of six categories. Through this reduction of character space, lineage-specific compositional heterogeneities are lessened—at the cost, however, of losing phylogenetic signal [67]. However, another advantage of Dayhoff recoding is a significant reduction of computation time needed to reach convergence.

The protein as well as the Dayhoff 6-state recoded dataset 2 were analyzed with PhyloBayes-MPI v1.7 [69,132], employing the CAT-GTR model, on the Linux cluster of the Leibniz Rechenzentrum (http://www.lrz.de) in Garching bei München, running 2 chains (each on 112 CPUs) each until reaching convergence, as estimated by using tracecomp and bpcomp programs of the PhyloBayes package (see PhyloBayes manual for details).

Furthermore, to evaluate the effect of using less-fitting site-homogeneous evolutionary models on the phylogenetic relationships of the Placozoa, we conducted a PhyloBayes-MPI analysis as above but with the GTR model (see for example [73], [68]), and also two maximum-likelihood analyses in RAxML: one with the GTR model using RAxML-NG v0.5.1b [139] [added options:—model PROTGTR+G+I—bs-trees 100—data-type AA] and one with the LG model using RAxML v8.2 [70] [added options: -f a -x 670 -m PROTGAMMAILG -p 220 -N 100]. The LG model was used as it was the best-fitting site-homogeneous model in 210 of the 212 gene partitions determined by ProtTest v3.4 [140]. Phylogenetic trees are shown as S15–S20 Figs.

## Supporting information

S1 Fig Ultrastructure of *Hoilungia hongkongensis*. The thin upper epithelium (A) essentially comprises flat cells (uec) with their cell body hanging underneath the surface, characteristic electron-dense granules (arrows in A) and, at times, large vacuoles. In the middle layer of the animal, numerous fiber cells (labeled “fc”) are identified, which contain cell type–specific mitochondrial complexes (labeled “mc”), large vacuoles with heterogeneous content, dense concrement vacuoles (labeled “cv”) and endosymbiotic bacteria in the endoplasmic reticulum (white arrowheads). In the lower epithelium, a few endocrine-like gland cells (labeled “gc”) are observed (B) among numerous epithelial cylinder cells (labeled “cc”; C) and lipophil cells (labeled “lc”; C, D). Each lipophil cell contains numerous middle-sized granules, one of which, called secretory granule (labeled “sg”), is abutting the lower membrane (E). Upper epithelium cells, gland cells, and lower epithelial cells are monociliated; the cilium is always located in a large ciliary pit (arrowheads in A, F). In both epithelia, cells are connected by apical junctions (see, e.g., arrows in E, F). The asterisk in (D) marks a long extension of a fiber cell. Scale bar in (D) (1 μm) also applies to (A-C). Scale bar in (F) (1 μm) also applies to (E**)**. ci, cilium.
(TIF)

S2 Fig Lava lamp plot of kmer coverage. The color code denotes the number of reads with a specific %GC and 31-bp kmer coverage. Heterozygous and homozygous coverage clouds show high counts at roughly 100x and 200x coverage, respectively.
(TIF)

S3 Fig Per base genome coverage. The gray area (81% of the assembly) marks bases of the reference assembly that are in the merged stage, with a peak at 260x coverage.
(TIF)

S4 Fig SNP histogram of genomic fractions. Plotted are SNP counts in the exonic, intronic, and intergenic genome fractions against the genome mapping coverage. The histogram shows that most of the genic (exonic and intronic) portions were merged (peak at approximately 250x coverage) and further indicates a very low number of false gene duplications caused by genome misassembly. SNP, single nucleotide polymorphism.
(TIF)

S5 Fig Length of syntenic blocks. Shown are numbers of genes in detected syntenic blocks between a reduced set of *Trichoplax adhaerens* scaffolds and *Hoilungia hongkongensis* contigs (blue circles; same set as used for collinearity analyses; see also Fig 2A & S5 Table) as well as between both whole genomes (red rectangles). Numbers of genes within blocks, as well as numbers of blocks are in the same order of magnitude, indicating that the reduced set is representative for full genomes.
(TIF)

S6 Fig Evaluation of codon saturation. Plotted are fractions of the full protein length for unchanged codons, as well as synonymous and nonsynonymous sites for 6,554 orthologs. dN1, dN2, and dN3 refer to nonsynonymous sites with single, double, and triple base change, respectively. Orthologs are sorted by increasing dN/dS ratio. Half of all orthologs have more than 40% unchanged sites (mean 45.1% ± 8.4%), and this value never drops below 16.5%. Third codon positions are thus never saturated, and the three orthologs with dN/dS > 1 are truly positively selected. dN/dS, nonsynonymous to synonymous nucleotide substitutions.
(TIF)

S7 Fig Comparison of inter- and intraspecific sequence divergence. Pairwise allelic (blue, green line) and interspecific (red, orange line) distances for 2,870 one-to-one orthologous genes. A significant fraction of orthologs have larger protein than CDS distance, but only three of these are, in fact, positively selected (reflected by dN/dS ratios > 1, gray line). Orthologs are sorted by increasing difference between the interspecific and the intraspecific protein sequence distance. Arrows mark the most prominent orthologs for which a high variation at the allelic level in *Hoilungia hongkongensis* is also mirrored by the sequence distance between *H*. *hongkongensis* and *Trichoplax adhaerens*. CDS, coding sequence; dN/dS, nonsynonymous to synonymous nucleotide substitutions.
(TIF)

S8 Fig Gene clustering and GO term enrichment analyses. (A) Gene clustering identified about half of both placozoan proteomes as one-to-one orthologs. A proportionally high number of proteins did not have any BLAST hits to the other placozoan species at all. Also, large fractions of placozoan-specific duplications were found in both species. (B) The high proportion of co-orthologs fall into four different categories (one-to-many, many-to-one, many-to-many, and many-to-zero). GO term–enrichment analyses (see small Venn diagrams) show that the one-to-many, as well as many-to-one bins, do not share enriched GO terms in the two species. In contrast, many-to-many bins share 80% of the top-5 GO terms, which is a validation of the clustering process. Many-to-zero co-orthologs show both shared and unique enriched GO-terms. The given results indicate that *Hoilungia hongkongensis* and *Trichoplax adhaerens* both have high percentages of individual gene duplications in various gene families. BP, biological process; CC, cellular component; GO, gene ontology; MF, molecular function.
(TIF)

S9 Fig 18S genetic distances in nonbilateria. Shown are mean group distances for different taxonomic ranks in the phyla Cnidaria, Ctenophora, and Porifera based on a full-length 18S rDNA alignment: between orders within classes, between families within orders, and between genera within families. The interspecific genetic distance between placozoans is shown on the right.
(TIF)

S10 Fig 28S genetic distances in nonbilateria. Shown are mean group distances for different taxonomic ranks in the phyla Cnidaria, Ctenophora, and Porifera, based on a full-length 28S rDNA alignment: between orders within classes, between families within orders, and between genera within families. The interspecific genetic distances between four placozoans are shown on the right.
(TIF)

S11 Fig 16S genetic distances in nonbilateria. Shown are mean group distances for different taxonomic ranks in the phyla Cnidaria, Ctenophora, and Porifera, based on a full-length 16S rDNA alignment: between orders within classes, between families within orders, and between genera within families. The interspecific genetic distances between 4 placozoans are shown on the right.
(TIF)

S12 Fig CO1 genetic distances in nonbilateria. Shown are mean group distances for different taxonomic ranks in the phyla Cnidaria, Ctenophora, and Porifera based on a full-length CO1 protein alignment: between orders within classes, between families within orders, and between genera within families. The interspecific genetic distances between 4 placozoans are shown on the right. CO1, cytochrome c oxidase subunit 1.
(TIF)

S13 Fig ND1 genetic distances in nonbilateria. Shown are mean group distances for different taxonomic ranks in the phyla Cnidaria, Ctenophora, and Porifera based on a full-length ND1 protein alignment: between orders within classes, between families within orders, and between genera within families. The interspecific genetic distances between 4 placozoans are shown on the right. ND1, NADH dehydrogenase subunit 1.
(TIF)

S14 Fig Summary of genetic distances of all markers. Shown are the means of all mean group distances for different taxonomic ranks in the nonbilaterian phyla Cnidaria, Ctenophora, and Porifera. The interspecific genetic distances between placozoans are shown on the right (yellow).
(TIF)

S15 Fig Phylogenetic tree based on the PhyloBayes analysis of dataset 2 (194 proteins). Posterior probability support is 1.0 unless otherwise noted. See S16 Fig for the underlying raw tree of the protein matrix. Schematic animal drawings derive from http://phylopic.org.
(TIF)

S16 Fig Full Bayesian (PhyloBayes) phylogeny of the dataset 2 protein matrix using CAT-GTR. CAT-GTR has been shown to have the best fit to multigene amino acid alignments [68,73].
(TIF)

S17 Fig Full Bayesian (PhyloBayes) phylogeny of the dataset 2 Dayhoff-6 recoded matrix using CAT-GTR. CAT-GTR has been shown to have the best fit to Dayhoff-6 recoded amino acid alignments [68]. Posterior Probabilities are given at nodes.
(TIF)

S18 Fig Full Bayesian (PhyloBayes) phylogeny of the dataset 2 protein matrix using GTR. GTR has been shown to have less fit to multigene amino acid alignments compared to CAT-GTR [68,73]. This phylogeny is provided here for comparative purposes only to display the effect of a less fitting evolutionary model on the tree topology (compare to S16 and S17 Figs). Posterior Probabilities are given at nodes.
(TIF)

S19 Fig Full Maximum Likelihood (RAxML) phylogeny of the dataset 2 protein matrix using GTR. GTR has been shown to have less fit to multigene amino acid alignments compared to CAT-GTR [68,73]. This phylogeny is provided here for comparative purposes only to display the effect of a less fitting evolutionary model on the tree topology (compare to Figs S16 and S17 Figs). Bootstrap support values are given at nodes. Clades with support of <70 have been collapsed and are drawn as a polytomy, due to a lack of confidence in those splits [141].
(TIF)

S20 Fig Full Maximum Likelihood (RAxML) phylogeny of the dataset 2 protein matrix using LG. The LG substitution model [141] has been shown to have less fit to multigene amino acid alignments compared to CAT-GTR [68,73]. This phylogeny is provided here for comparative purposes only to display the effect of a less fitting evolutionary model on the tree topology (compare to Figs S16 and S17 Figs). Bootstrap support values are given at nodes. Clades with support of <70 have been collapsed and are drawn as a polytomy because of a lack of confidence in those splits [142].
(TIF)

S21 Fig Gene occupancy matrix for dataset 2 used for phylogenomic analyses. Plotted are all 194 proteins, sorted based on the phylogenetic tree given in Fig 7. White space indicates missing sequence information for a protein. Color intensity is related to the percentage of gene completeness in partial proteins. Note that most of the proteins in the matrix are complete.
(TIF)

S1 Table *Hoilungia hongkongensis* genome assembly statistics. *H*. *hongkongensis* genome assembly and annotation statistics in comparison to *Trichoplax adhaerens*. n/a, not available.
(XLSX)

S2 Table Summary of *Hoilungia hongkongensis* SNP counts. Shown are summaries from the genomic and transcriptomic (M153E-2 strain) datasets. SNP, single nucleotide polymorphism.
(XLSX)

S3 Table *Hoilungia hongkongensis* read- and transcript-mapping statistics. (XLSX)

S4 Table Results of the *Hoilungia hongkongensis* BUSCO v3 searches. (XLSX)

S5 Table Results of collinearity analysis. Collinearity between the 10 largest *Trichoplax adhaerens* (labeled “*T*.*a*.”) scaffolds and the associated 144 *Hoilungia hongkongensis* (labeled “*H*.*h*.”) supercontigs >100 Kb.
(XLSX)

S6 Table Samples used to generate the conspecificity matrix. Shown are all isolates, including their origin. The provided isolate ID was used in Fig 3 in addition to the haplotype. AS, aquarium sample.
(XLSX)

S7 Table Transcriptomic and genomic data resources. Used data for genetic distance calculations and phylogenetic inferences. A Trinity transcriptome assembly has been generated for each species with a given SRA accession number. Otherwise, transcriptomes and/or protein sequences from genome annotations were used from the reference. SRA, Sequence Read Archive.
(XLSX)

S8 Table List of OTUs. Listed are all OTUs used for phylogenetic analyses (dataset 2, as specified). The first species of each participated most. OTU, operational taxonomic unit.
(XLSX)

S9 Table Datasets used for distance calculation and phylogenetic inferences. Summary of protein datasets used for distance calculations (dataset 1) and phylogenetic inferences (dataset 2). Matrix length is 37,838 amino acid characters (dataset 1) and 35,799 (dataset 2), respectively.
(XLSX)

S10 Table Genetic distances (%) between the two placozoan species in comparison to Cnidaria. Shown are averages of all mean genetic distances (±SD) between genera within families and families within orders for the Cnidaria, as well as genetic distances between *Hoilungia hongkongensis* (labeled “*H*. *h*.”) and *Trichoplax adhaerens* (labeled “*T*. *a*.”). The number of all mean pairwise distances used to calculate the average is given in parentheses.
(XLSX)

S11 Table Accession numbers of mitochondrial genomes used. (XLSX)

S1 Data *Hoilungia hongkongensis* gene ontology annotation table. (XLSX)

S2 Data *Hoilungia hongkongensis* annotation table (own script). (XLSX)

S3 Data *Trichoplax adhaerens* GO annotation table. (XLSX)

S4 Data Lists of top-5 enriched gene ontology terms. (XLSX)

S5 Data Full lists of enriched gene ontology terms. (XLSX)

S6 Data Divergent copy numbers in *Hoilungia hongkongensis* and *Trichoplax adhaerens* in genes tagged as GPCR signaling. GPCR, G-protein-coupled receptor.
(XLSX)

S7 Data Orthologs between *Hoilungia hongkongensis* and *Trichoplax adhaerens* with dN/dS > 1.0. dN/dS, nonsynonymous to synonymous nucleotide substitutions.
(XLSX)

S8 Data Calculated pairwise distances of 212 proteins for the Placozoa, Porifera, Cnidaria, Ctenophora, and Chordata. (XLSX)

## Abbreviations

CDS: coding sequence
CM: conspecificity matrix
CO1: cytochrome c oxidase subunit 1
dN/dS: nonsynonymous to synonymous nucleotide substitutions
FPKM: fragments per kilobase million
GO: gene ontology
GPCR: G-protein coupled receptor
HMM: hidden Markov model
ITS2: internal transcribed spacer 2
MCL: Markov cluster
ND1: NADH dehydrogenase subunit 1
rDNA: ribosomal DNA
SNP: single nucleotide polymorphism
TPM: transcripts per kilobase million
