# Correction: Comparative genomics and the nature of placozoan species

**DOI:** 10.1371/journal.pbio.3000032

**Published:** 2018-09-19

**Authors:** Michael Eitel, Warren R. Francis, Frédérique Varoqueaux, Jean Daraspe, Hans-Jürgen Osigus, Stefan Krebs, Sergio Vargas, Helmut Blum, Gray A. Williams, Bernd Schierwater, Gert Wörheide

This published work and the nomenclatural acts it contains have been registered in ZooBank (http://zoobank.org/), the online registration system for the International Code of Zoological Nomenclature (ICZN). The ZooBank LSIDs (Life Science Identifiers) can be resolved and the associated information viewed through any standard web browser by the following URL:

http://zoobank.org/NomenclaturalActs/2B0765C1-D939-48FD-B573-5B3F381817B7

The LSID for this publication is:

urn:lsid:zoobank.org:act:2B0765C1-D939-48FD-B573-5B3F381817B7

The formal taxonomic diagnosis has been amended:

Type Genus: *Hoilungia*, nov. gen., Eitel, Schierwater & Wörheide

LSIDurn:lsid:zoobank.org:act:BBE660BA-5094-4F39-9324-096C1ED88B9D

Type species: *H. hongkongensis*, nov. spec., Eitel, Schierwater & Wörheide

urn:lsid:zoobank.org:act:2B0765C1-D939-48FD-B573-5B3F381817B7
