# Supplementary figures and images for: Comparative genomics and the nature of placozoan species
Source: PLoS Biol. 2018 Jul 31;16(7):e2005359. doi: 10.1371/journal.pbio.2005359 (PMC6067683; doi:10.1371/journal.pbio.2005359)

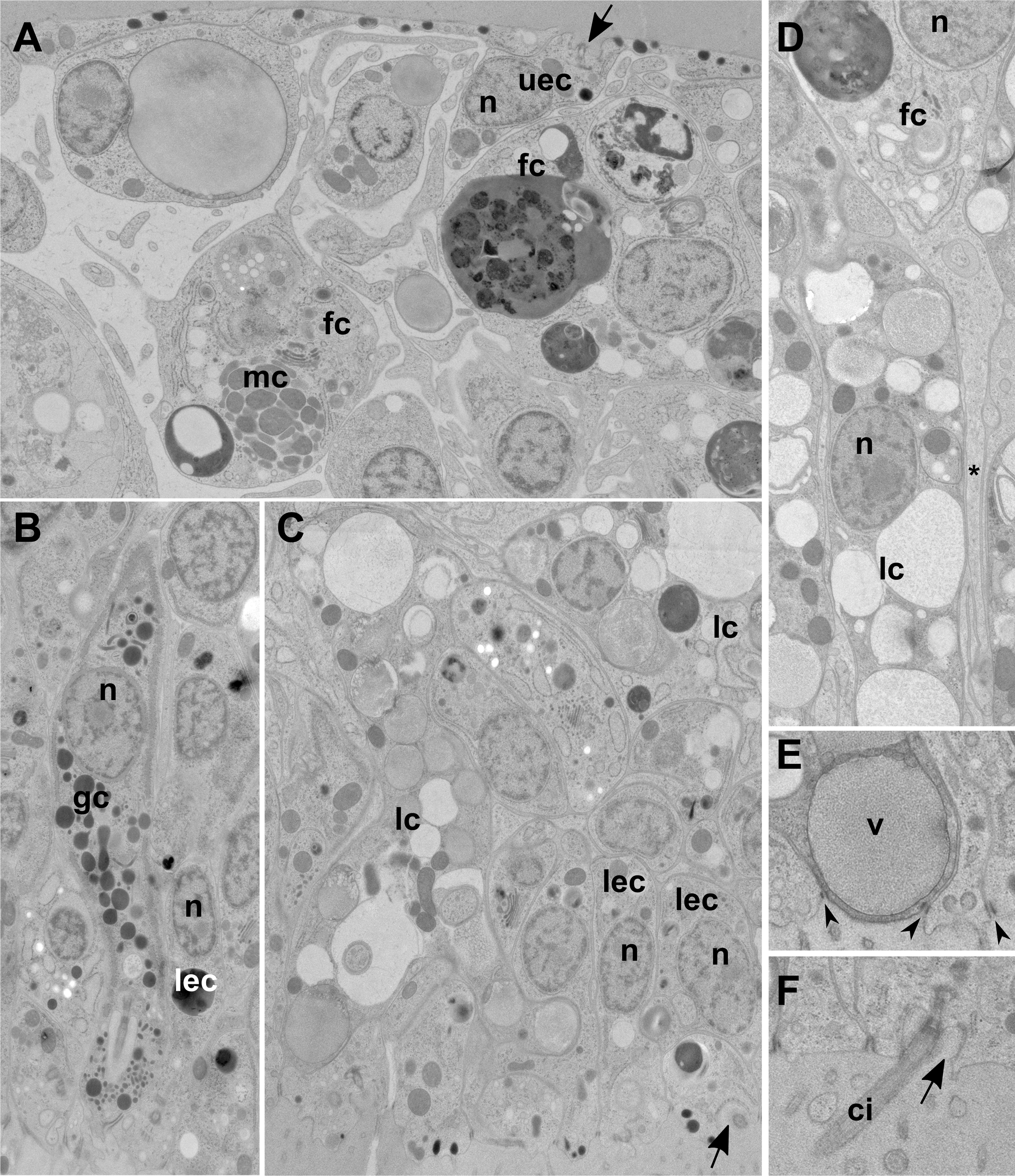

Supplement: S1 Fig — The thin upper epithelium (A) essentially comprises flat cells (uec) with their cell body hanging underneath the surface, characteristic electron-dense granules (arrows in A) and, at times, large vacuoles. In the middle layer of the animal, numerous fiber cells (labeled “fc”) are identified, which contain cell type–specific mitochondrial complexes (labeled “mc”), large vacuoles with heterogeneous content, dense concrement vacuoles (labeled “cv”) and endosymbiotic bacteria in the endoplasmic reticulum (white arrowheads). In the lower epithelium, a few endocrine-like gland cells (labeled “gc”) are observed (B) among numerous epithelial cylinder cells (labeled “cc”; C) and lipophil cells (labeled “lc”; C, D). Each lipophil cell contains numerous middle-sized granules, one of which, called secretory granule (labeled “sg”), is abutting the lower membrane (E). Upper epithelium cells, gland cells, and lower epithelial cells are monociliated; the cilium is always located in a large ciliary pit (arrowheads in A, F). In both epithelia, cells are connected by apical junctions (see, e.g., arrows in E, F). The asterisk in (D) marks a long extension of a fiber cell. Scale bar in (D) (1 μm) also applies to (A-C). Scale bar in (F) (1 μm) also applies to (E). ci, cilium. (TIF) [file pbio.2005359.s001.tif]

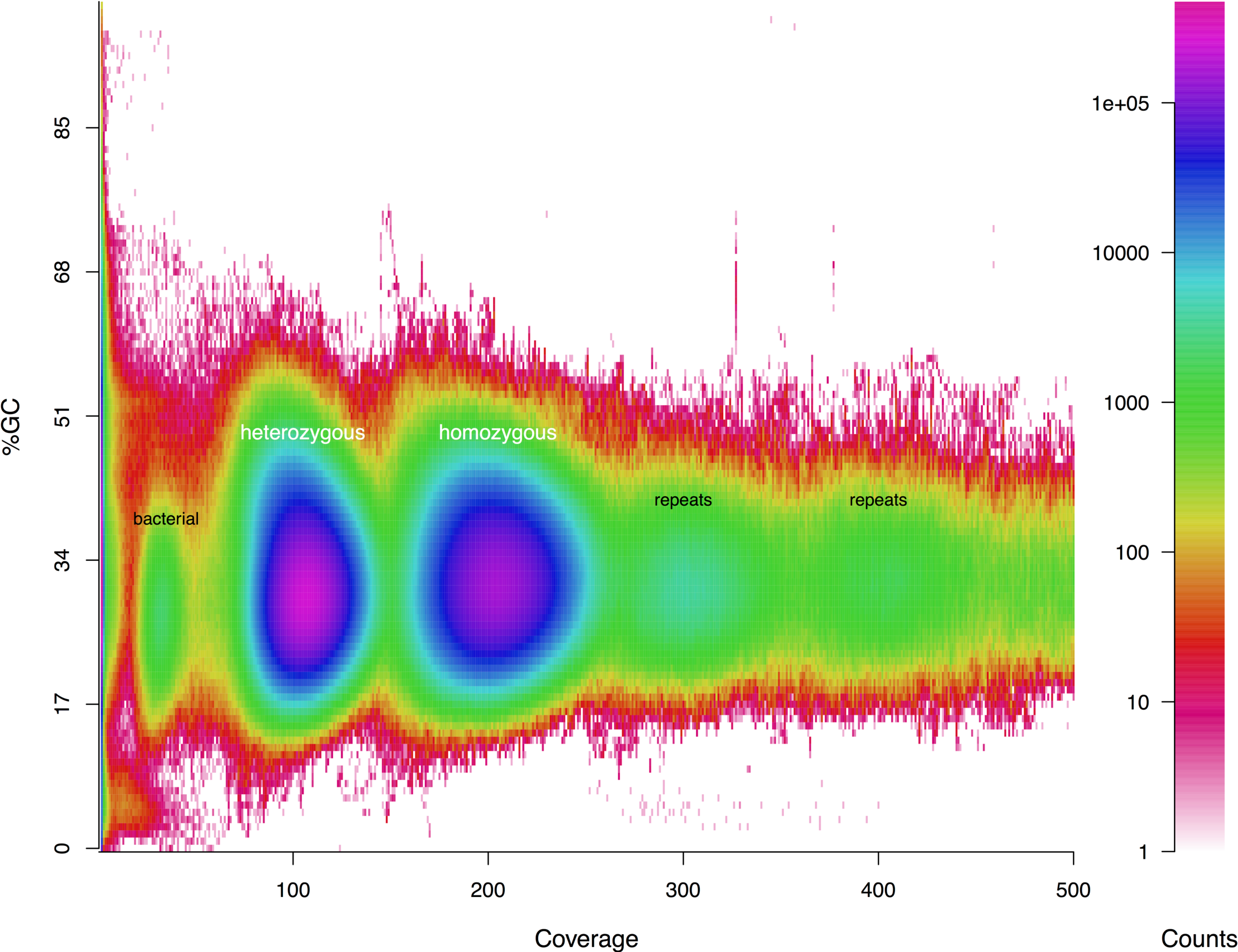

Supplement: S2 Fig — The color code denotes the number of reads with a specific %GC and 31-bp kmer coverage. Heterozygous and homozygous coverage clouds show high counts at roughly 100x and 200x coverage, respectively. (TIF) [file pbio.2005359.s002.tif]

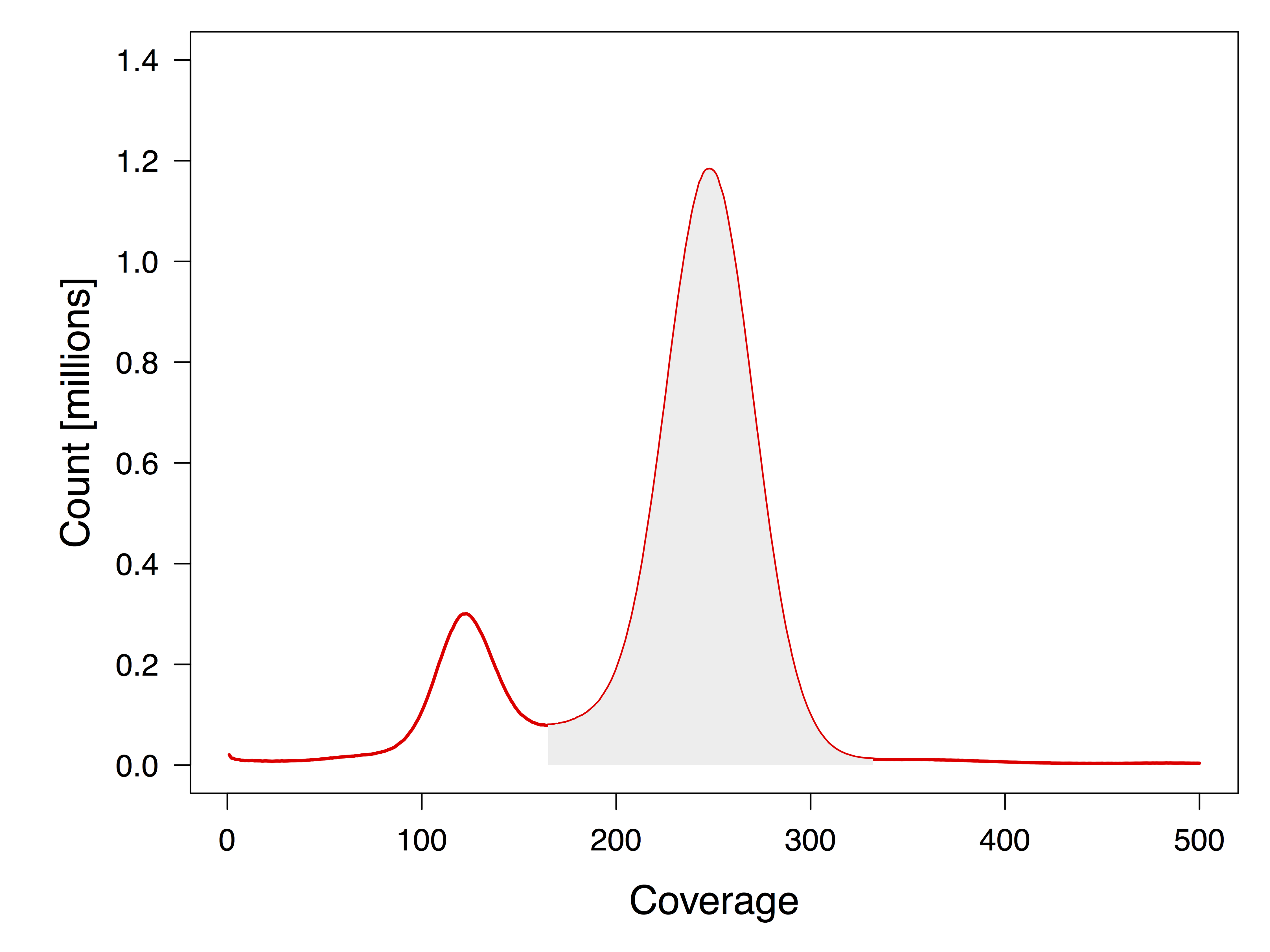

Supplement: S3 Fig — The gray area (81% of the assembly) marks bases of the reference assembly that are in the merged stage, with a peak at 260x coverage. (TIF) [file pbio.2005359.s003.tif]

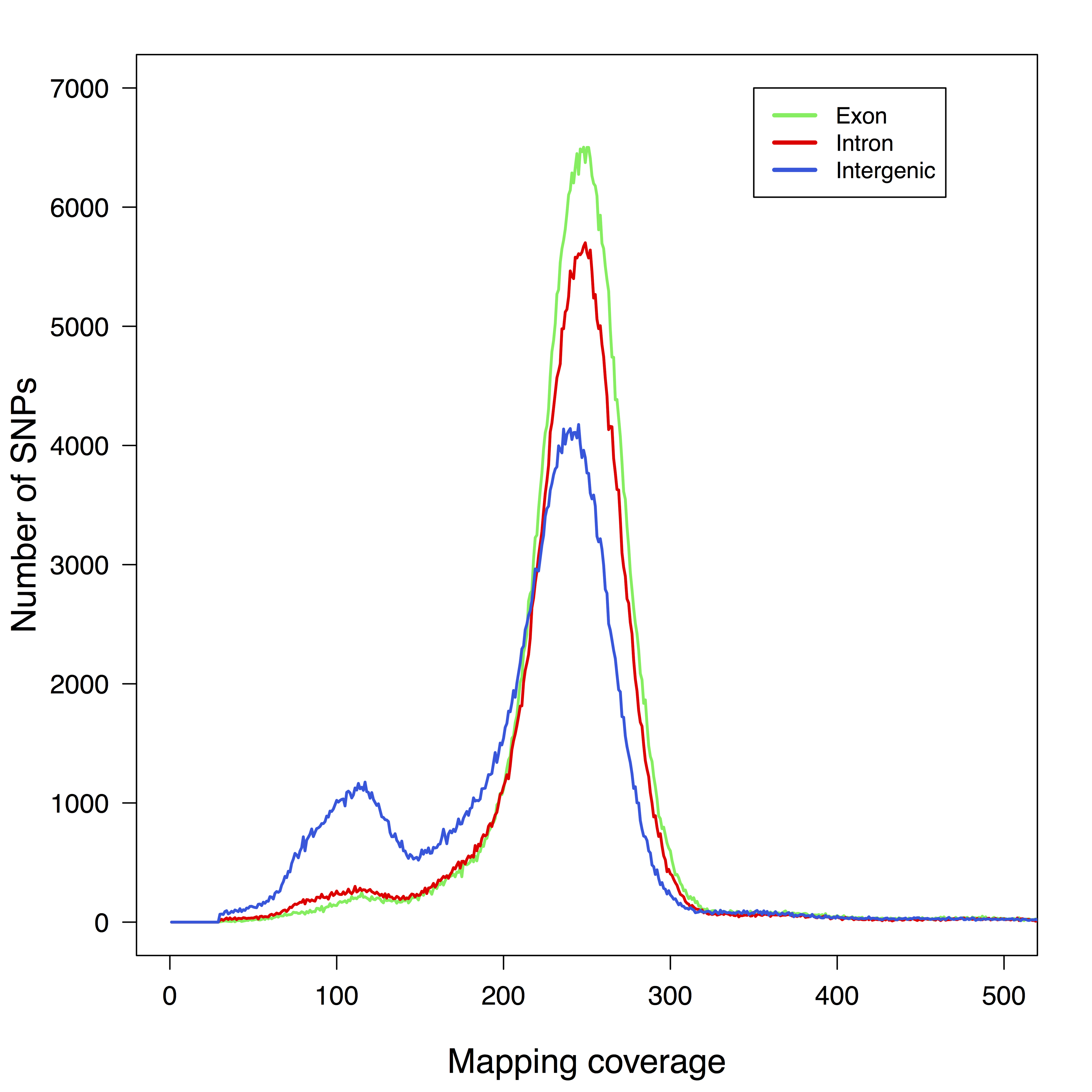

Supplement: S4 Fig — Plotted are SNP counts in the exonic, intronic, and intergenic genome fractions against the genome mapping coverage. The histogram shows that most of the genic (exonic and intronic) portions were merged (peak at approximately 250x coverage) and further indicates a very low number of false gene duplications caused by genome misassembly. SNP, single nucleotide polymorphism. (TIF) [file pbio.2005359.s004.tif]

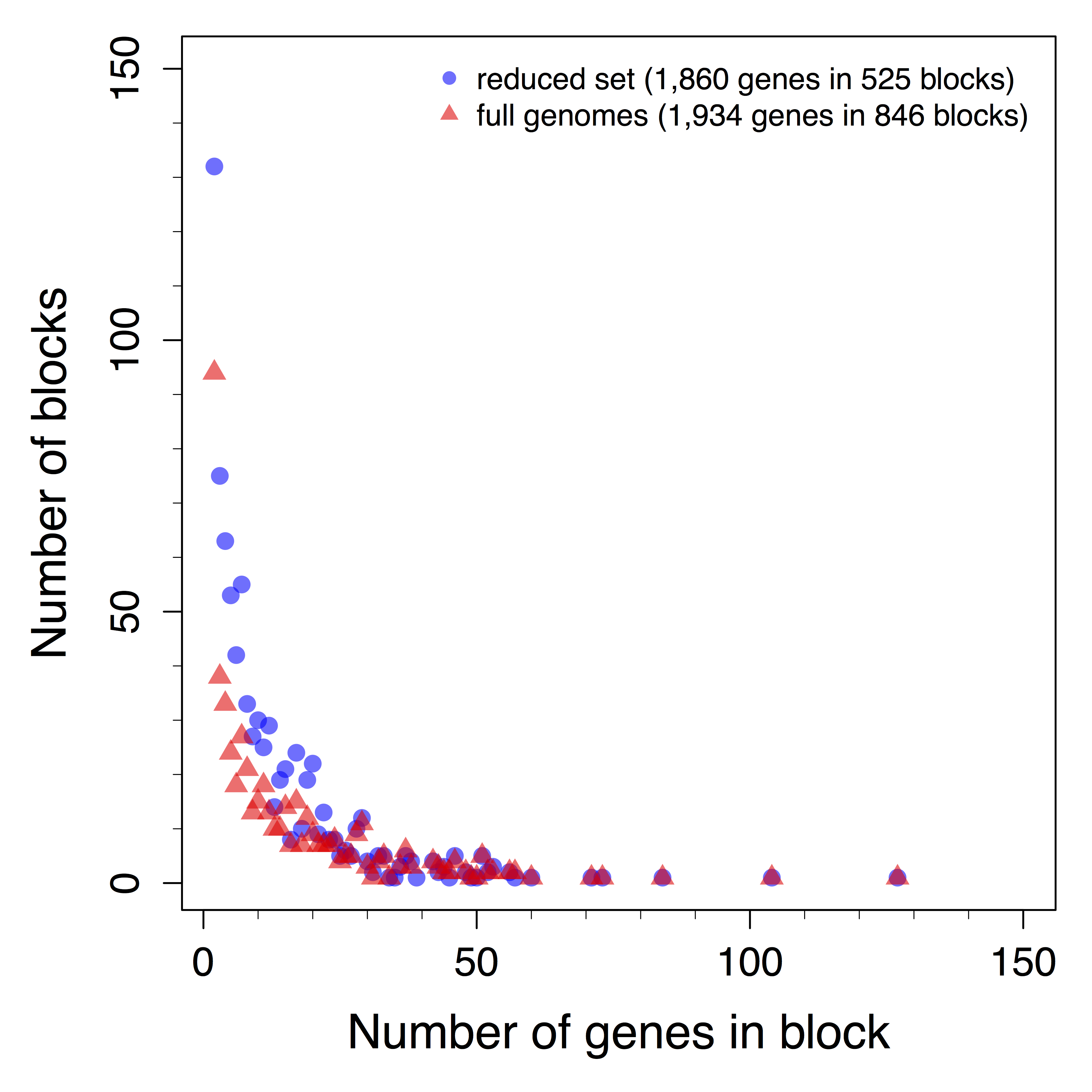

Supplement: S5 Fig — Shown are numbers of genes in detected syntenic blocks between a reduced set of Trichoplax adhaerens scaffolds and Hoilungia hongkongensis contigs (blue circles; same set as used for collinearity analyses; see also Fig 2A & S5 Table) as well as between both whole genomes (red rectangles). Numbers of genes within blocks, as well as numbers of blocks are in the same order of magnitude, indicating that the reduced set is representative for full genomes. (TIF) [file pbio.2005359.s005.tif]

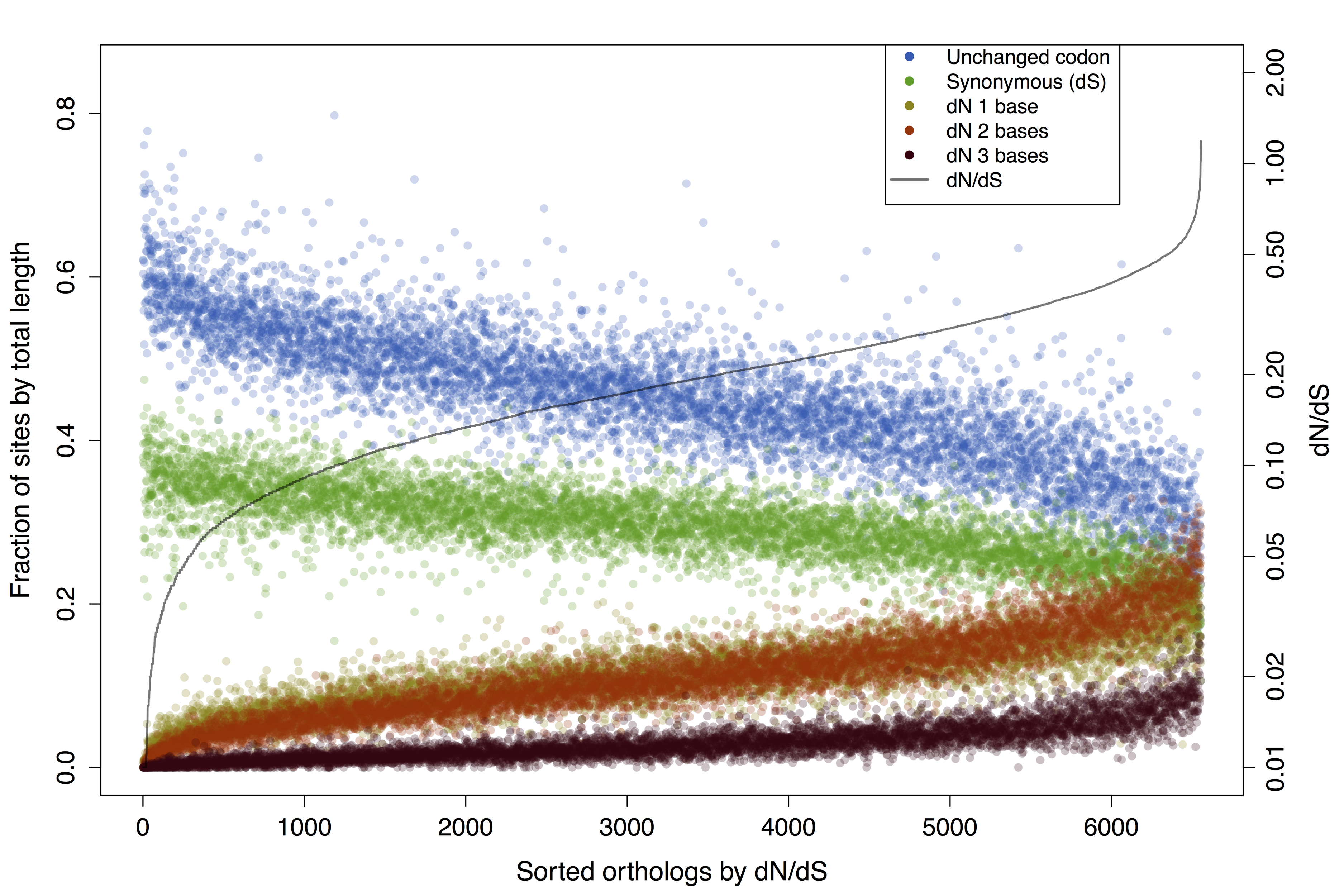

Supplement: S6 Fig — Plotted are fractions of the full protein length for unchanged codons, as well as synonymous and nonsynonymous sites for 6,554 orthologs. dN1, dN2, and dN3 refer to nonsynonymous sites with single, double, and triple base change, respectively. Orthologs are sorted by increasing dN/dS ratio. Half of all orthologs have more than 40% unchanged sites (mean 45.1% ± 8.4%), and this value never drops below 16.5%. Third codon positions are thus never saturated, and the three orthologs with dN/dS > 1 are truly positively selected. dN/dS, nonsynonymous to synonymous nucleotide substitutions. (TIF) [file pbio.2005359.s006.tif]

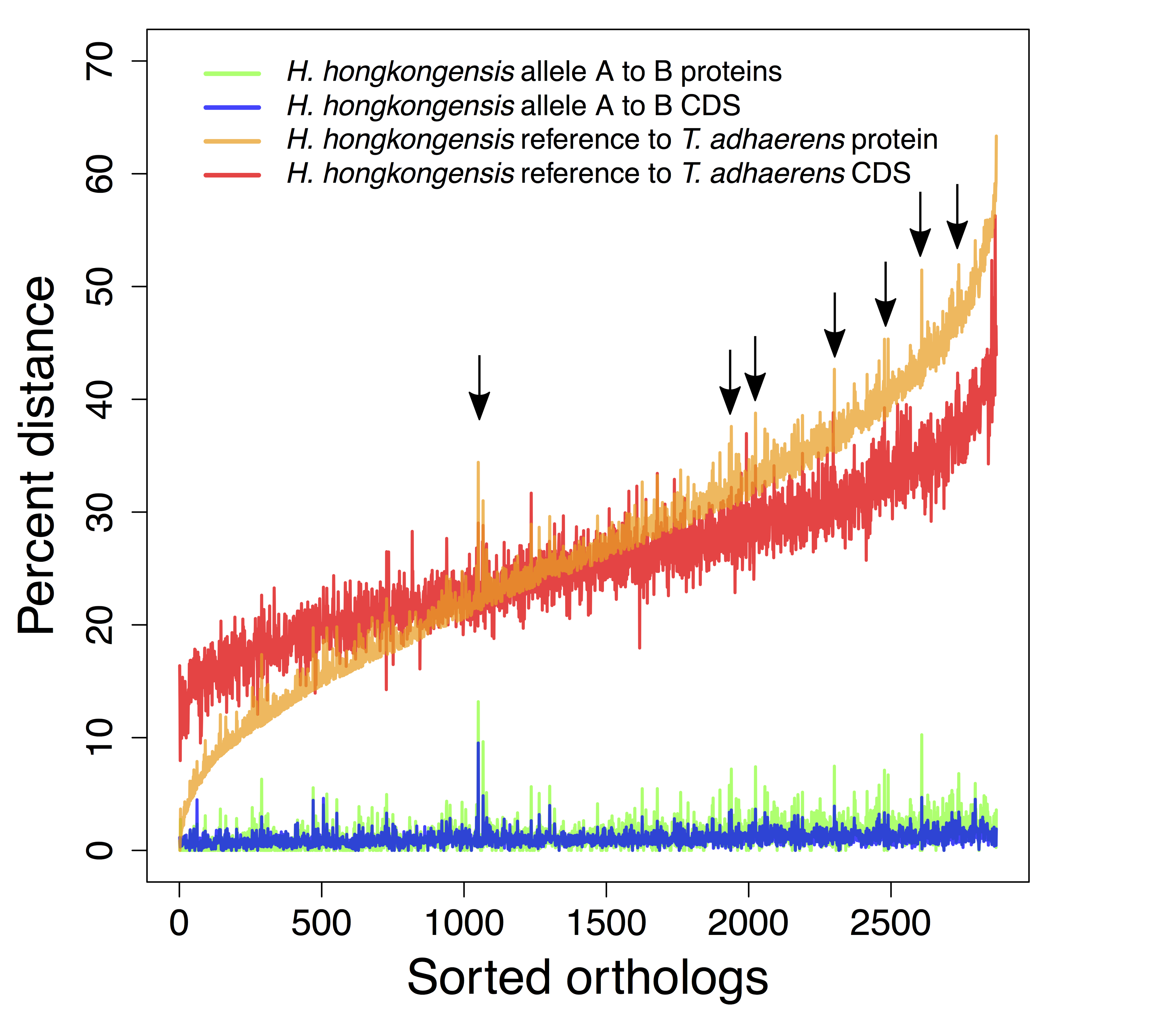

Supplement: S7 Fig — Pairwise allelic (blue, green line) and interspecific (red, orange line) distances for 2,870 one-to-one orthologous genes. A significant fraction of orthologs have larger protein than CDS distance, but only three of these are, in fact, positively selected (reflected by dN/dS ratios > 1, gray line). Orthologs are sorted by increasing difference between the interspecific and the intraspecific protein sequence distance. Arrows mark the most prominent orthologs for which a high variation at the allelic level in Hoilungia hongkongensis is also mirrored by the sequence distance between H. hongkongensis and Trichoplax adhaerens. CDS, coding sequence; dN/dS, nonsynonymous to synonymous nucleotide substitutions. (TIF) [file pbio.2005359.s007.tif]

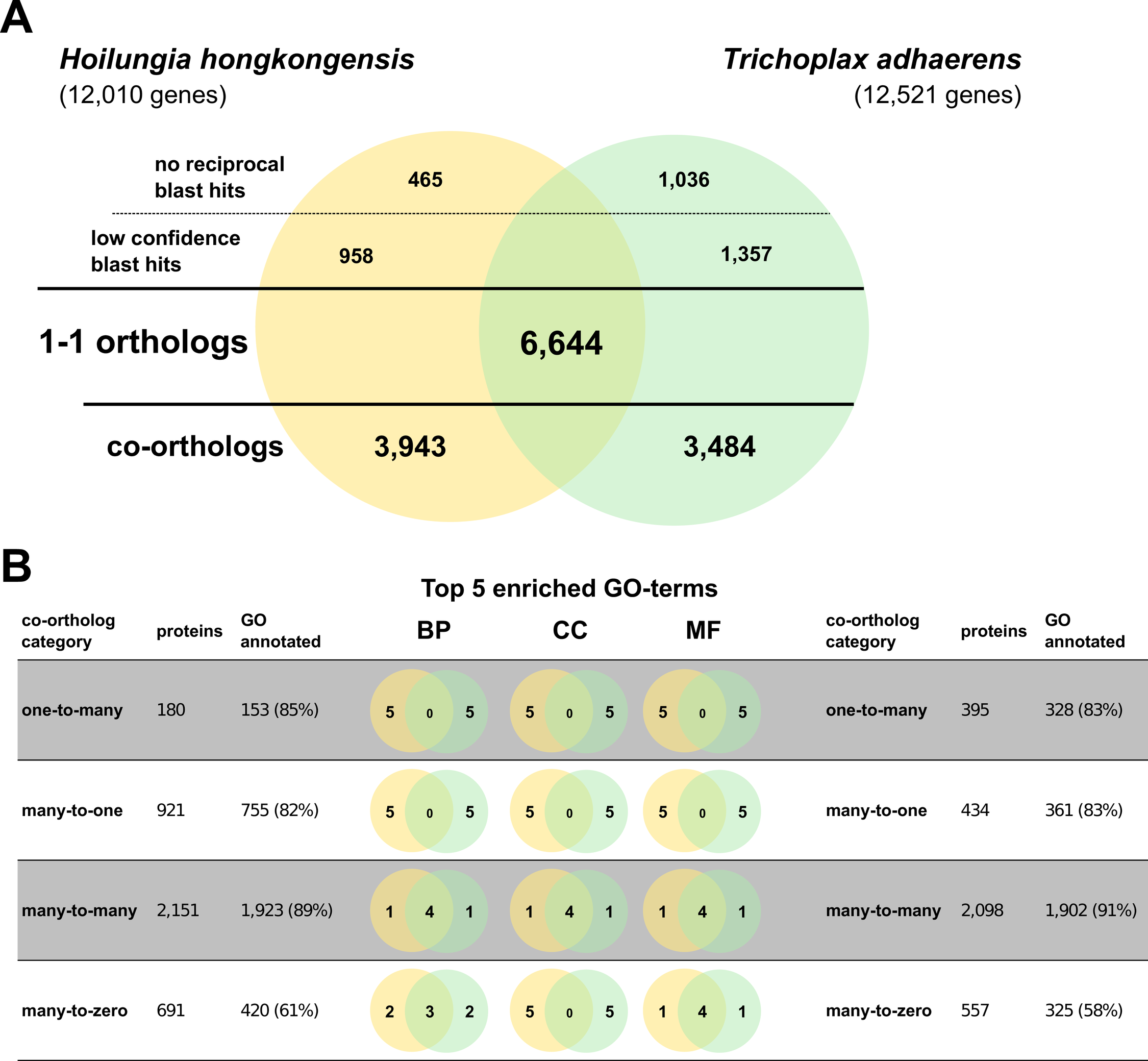

Supplement: S8 Fig — (A) Gene clustering identified about half of both placozoan proteomes as one-to-one orthologs. A proportionally high number of proteins did not have any BLAST hits to the other placozoan species at all. Also, large fractions of placozoan-specific duplications were found in both species. (B) The high proportion of co-orthologs fall into four different categories (one-to-many, many-to-one, many-to-many, and many-to-zero). GO term–enrichment analyses (see small Venn diagrams) show that the one-to-many, as well as many-to-one bins, do not share enriched GO terms in the two species. In contrast, many-to-many bins share 80% of the top-5 GO terms, which is a validation of the clustering process. Many-to-zero co-orthologs show both shared and unique enriched GO-terms. The given results indicate that Hoilungia hongkongensis and Trichoplax adhaerens both have high percentages of individual gene duplications in various gene families. BP, biological process; CC, cellular component; GO, gene ontology; MF, molecular function. (TIF) [file pbio.2005359.s008.tif]

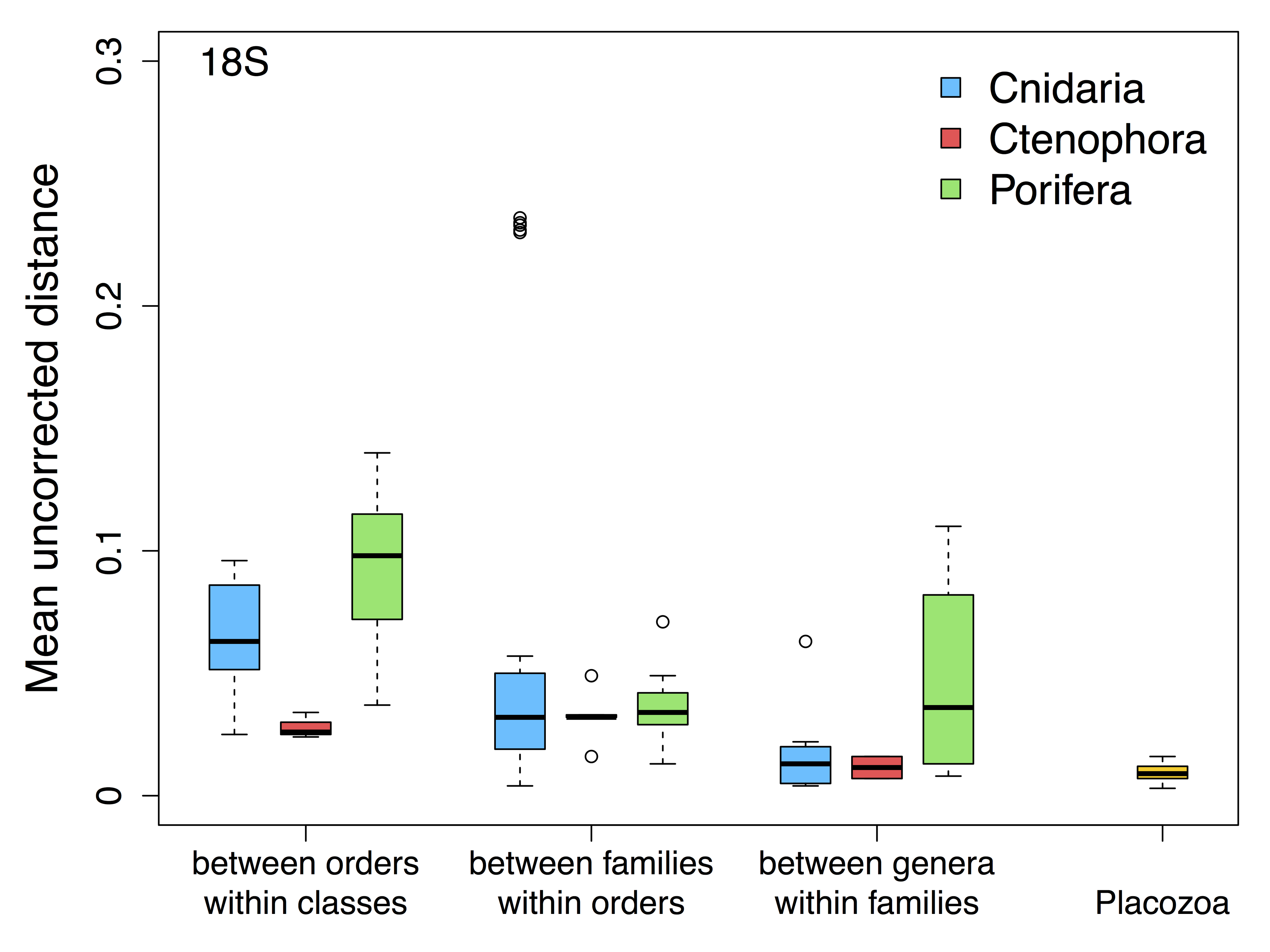

Supplement: S9 Fig — Shown are mean group distances for different taxonomic ranks in the phyla Cnidaria, Ctenophora, and Porifera based on a full-length 18S rDNA alignment: between orders within classes, between families within orders, and between genera within families. The interspecific genetic distance between placozoans is shown on the right. (TIF) [file pbio.2005359.s009.tif]

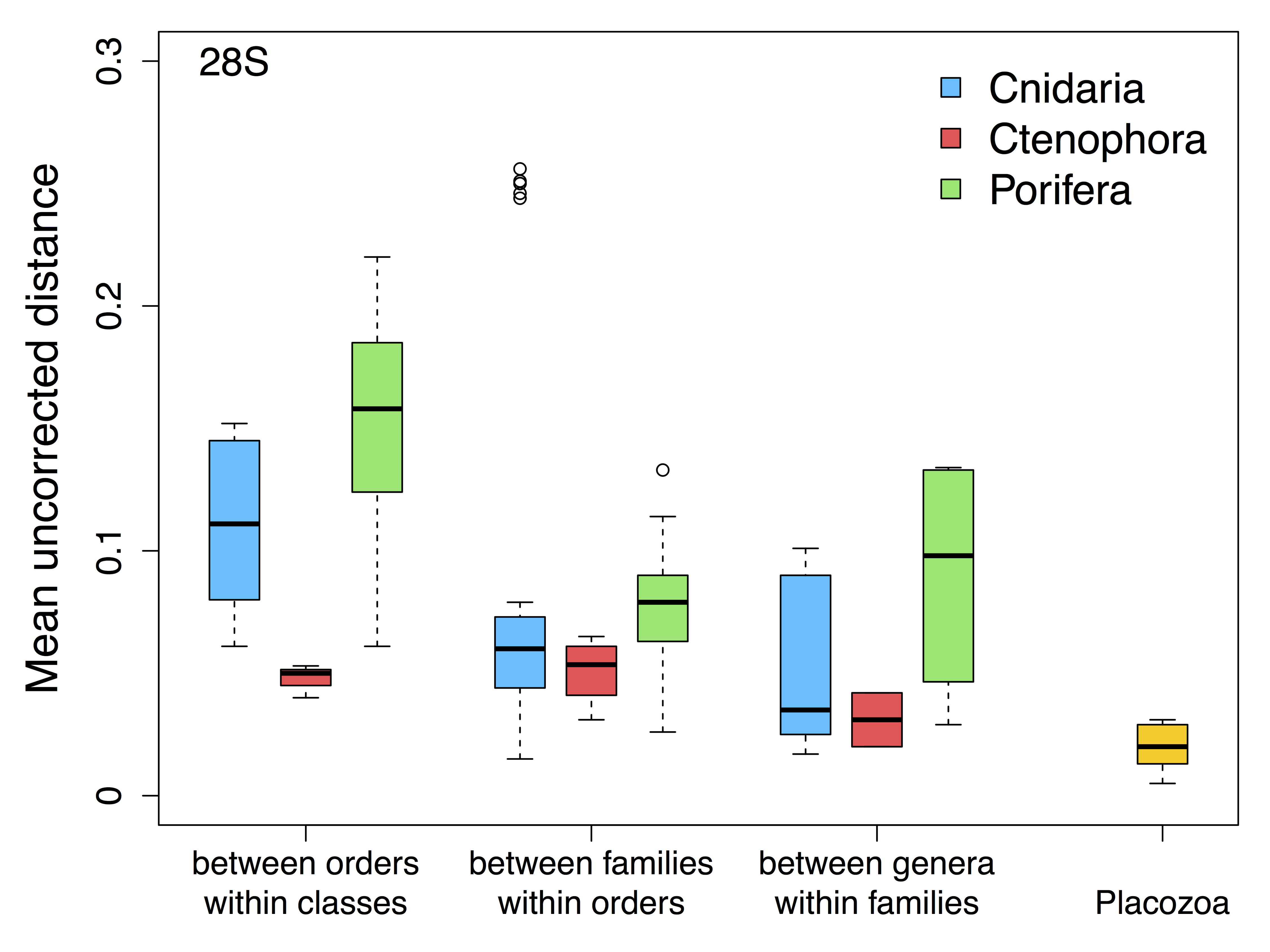

Supplement: S10 Fig — Shown are mean group distances for different taxonomic ranks in the phyla Cnidaria, Ctenophora, and Porifera, based on a full-length 28S rDNA alignment: between orders within classes, between families within orders, and between genera within families. The interspecific genetic distances between four placozoans are shown on the right. (TIF) [file pbio.2005359.s010.tif]

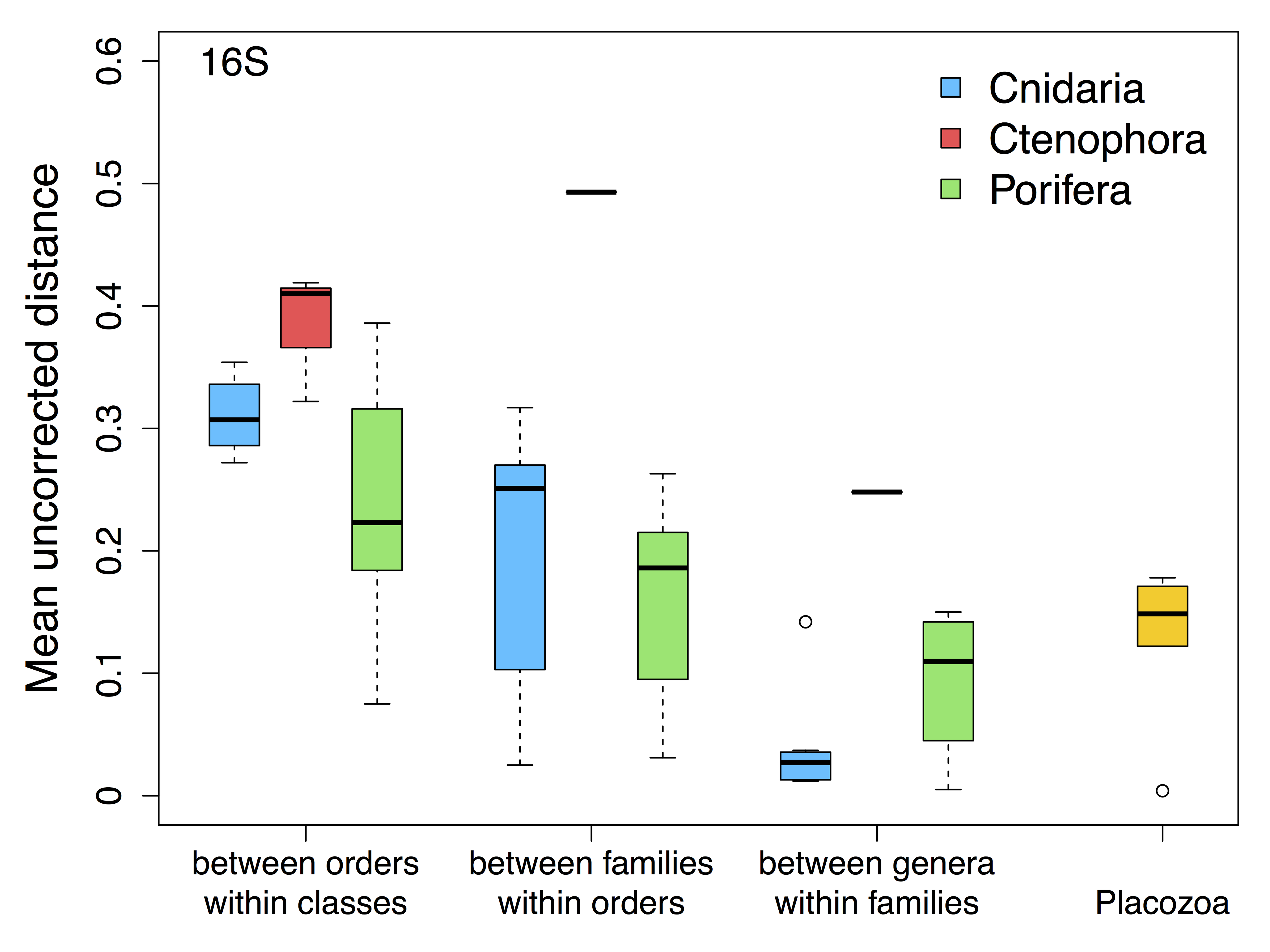

Supplement: S11 Fig — Shown are mean group distances for different taxonomic ranks in the phyla Cnidaria, Ctenophora, and Porifera, based on a full-length 16S rDNA alignment: between orders within classes, between families within orders, and between genera within families. The interspecific genetic distances between 4 placozoans are shown on the right. (TIF) [file pbio.2005359.s011.tif]

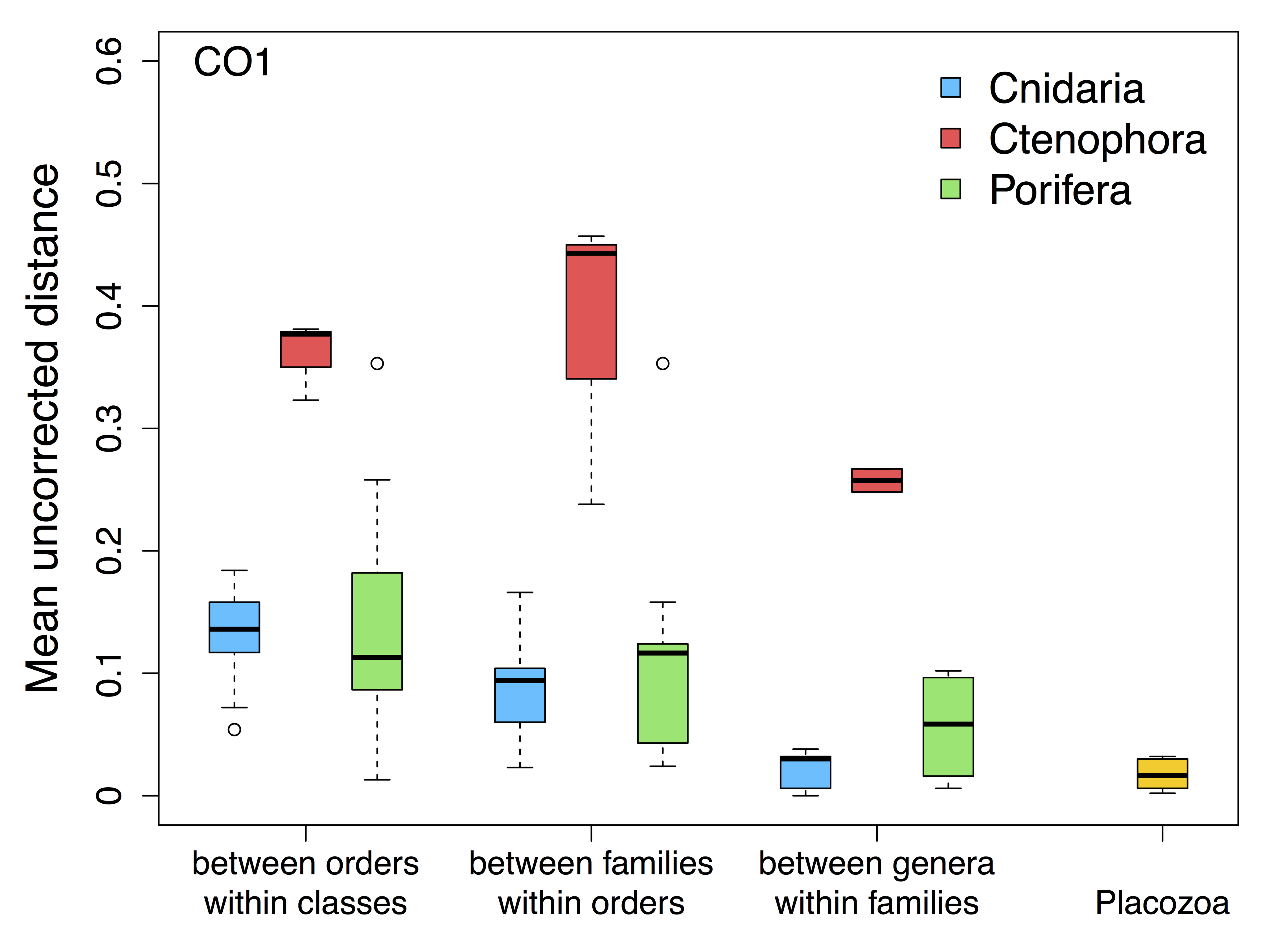

Supplement: S12 Fig — Shown are mean group distances for different taxonomic ranks in the phyla Cnidaria, Ctenophora, and Porifera based on a full-length CO1 protein alignment: between orders within classes, between families within orders, and between genera within families. The interspecific genetic distances between 4 placozoans are shown on the right. CO1, cytochrome c oxidase subunit 1. (TIF) [file pbio.2005359.s012.tif]

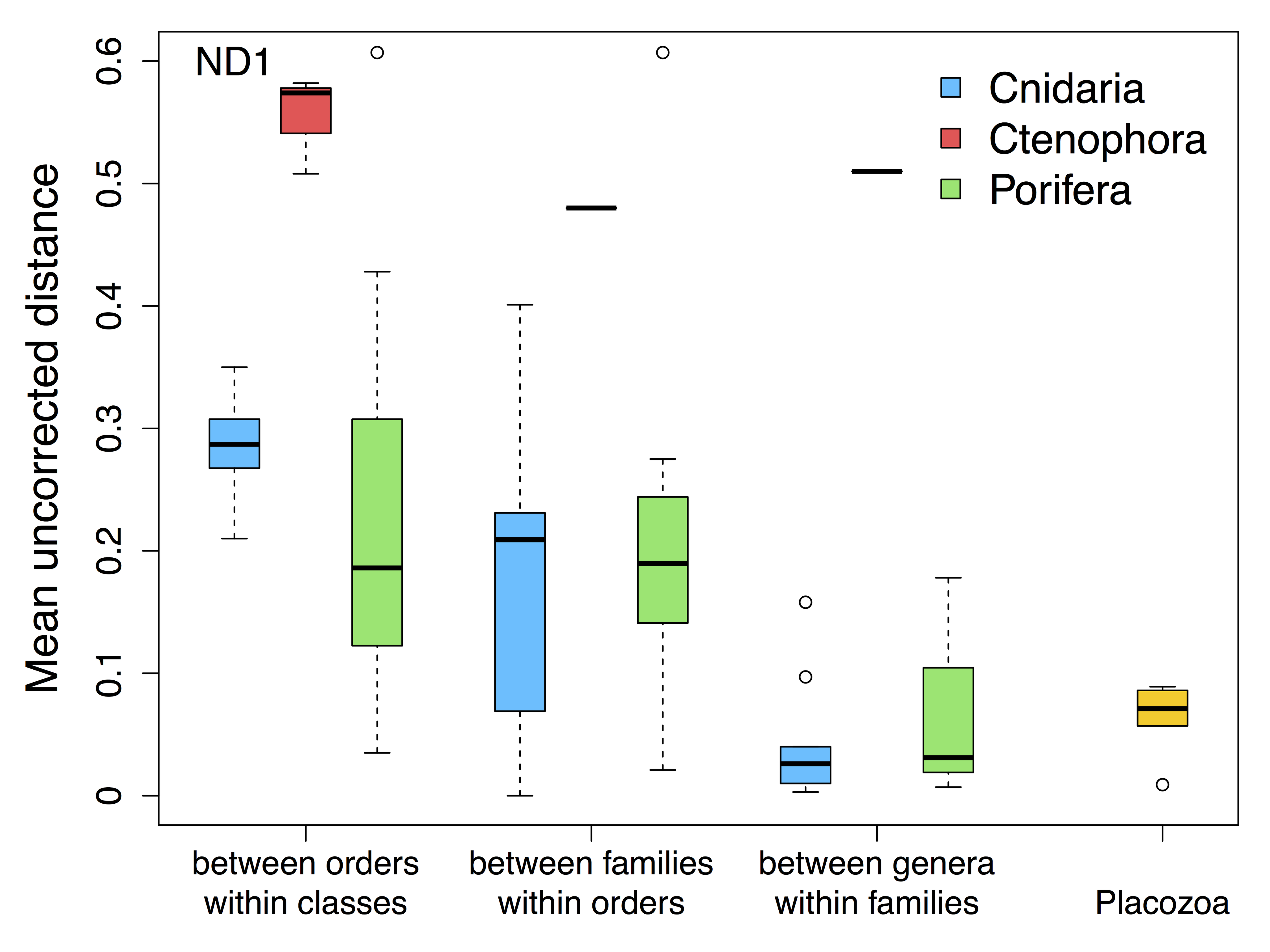

Supplement: S13 Fig — Shown are mean group distances for different taxonomic ranks in the phyla Cnidaria, Ctenophora, and Porifera based on a full-length ND1 protein alignment: between orders within classes, between families within orders, and between genera within families. The interspecific genetic distances between 4 placozoans are shown on the right. ND1, NADH dehydrogenase subunit 1. (TIF) [file pbio.2005359.s013.tif]

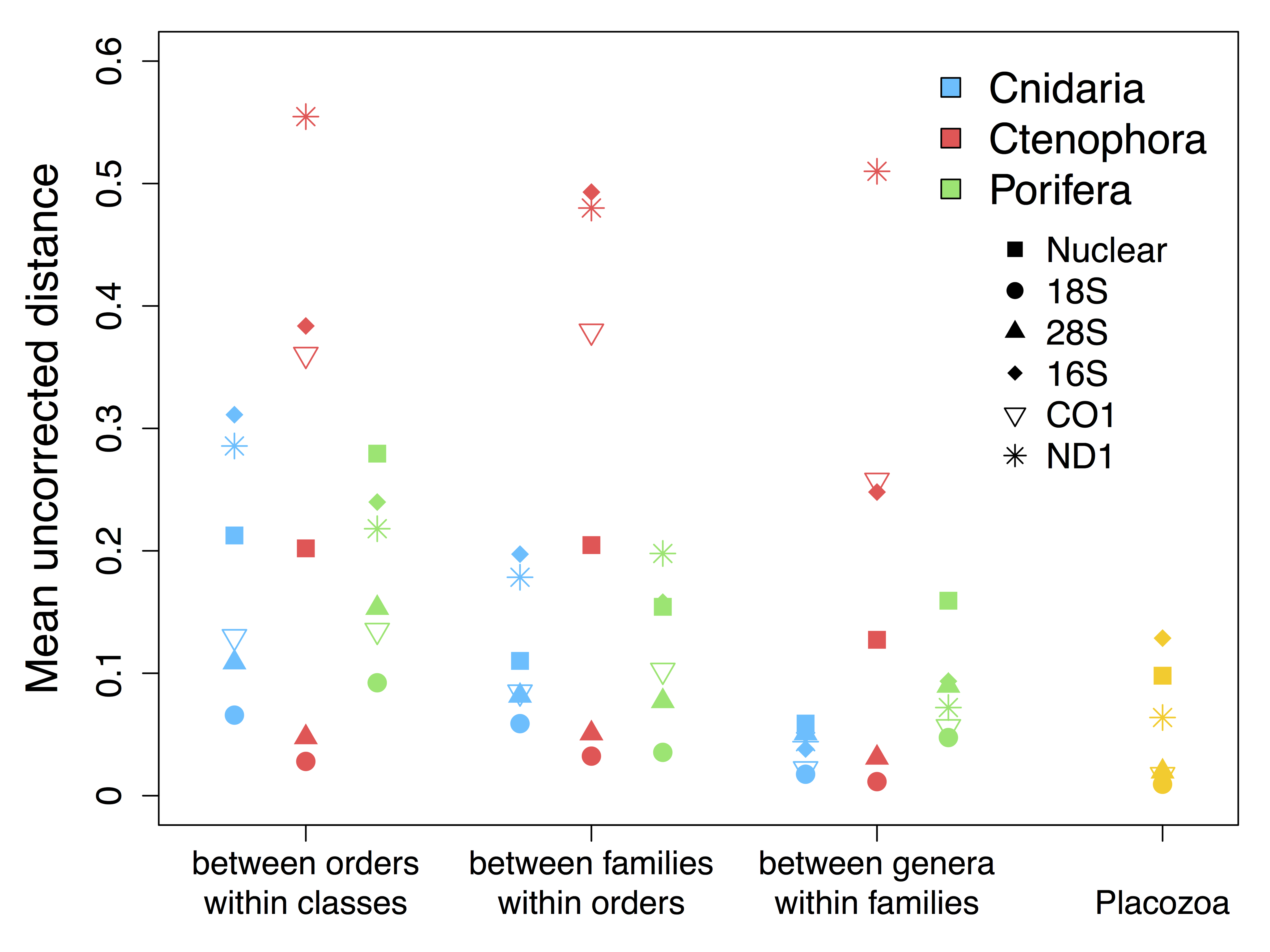

Supplement: S14 Fig — Shown are the means of all mean group distances for different taxonomic ranks in the nonbilaterian phyla Cnidaria, Ctenophora, and Porifera. The interspecific genetic distances between placozoans are shown on the right (yellow). (TIF) [file pbio.2005359.s014.tif]

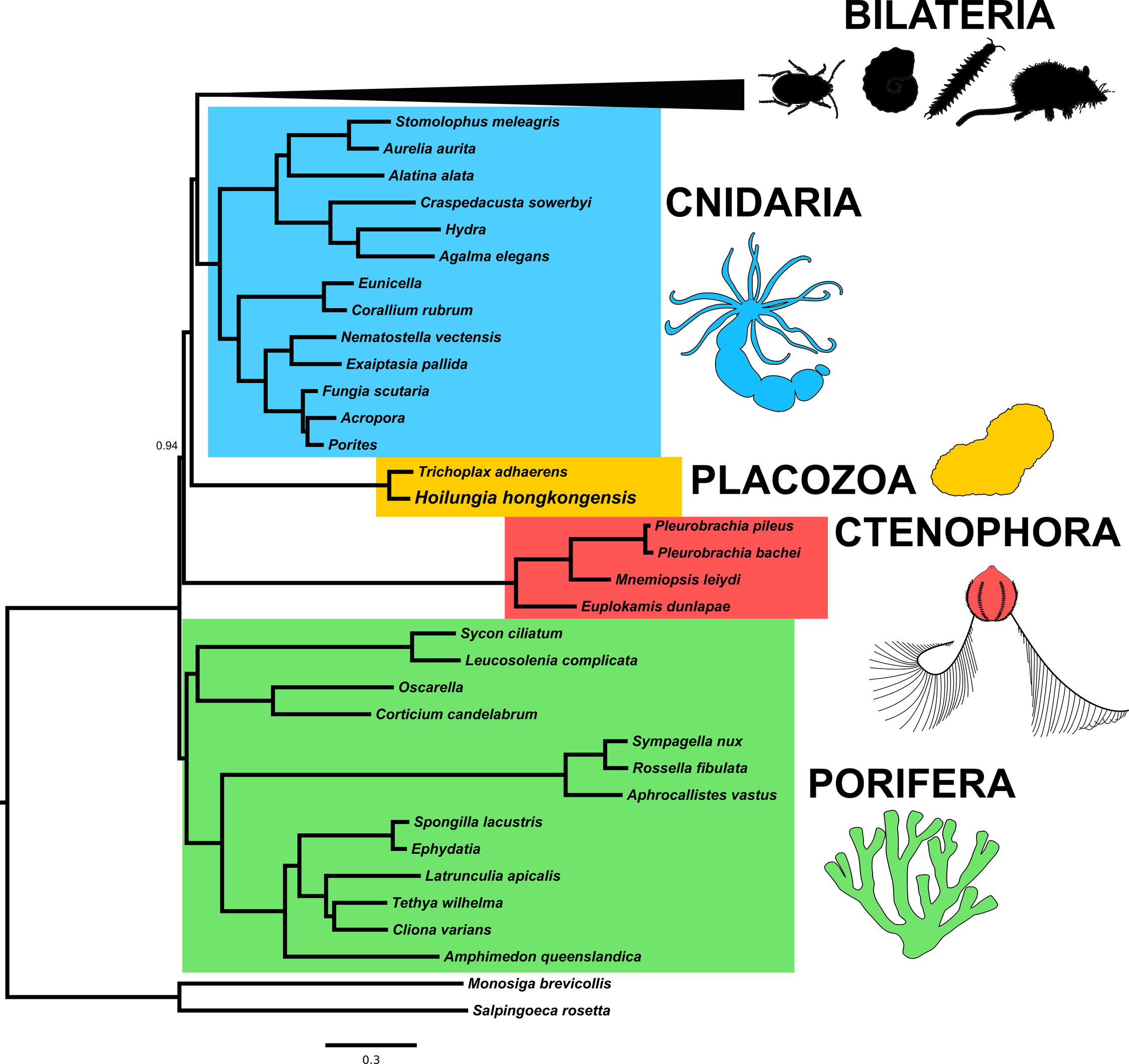

Supplement: S15 Fig — Posterior probability support is 1.0 unless otherwise noted. See S16 Fig for the underlying raw tree of the protein matrix. Schematic animal drawings derive from http://phylopic.org. (TIF) [file pbio.2005359.s015.tif]

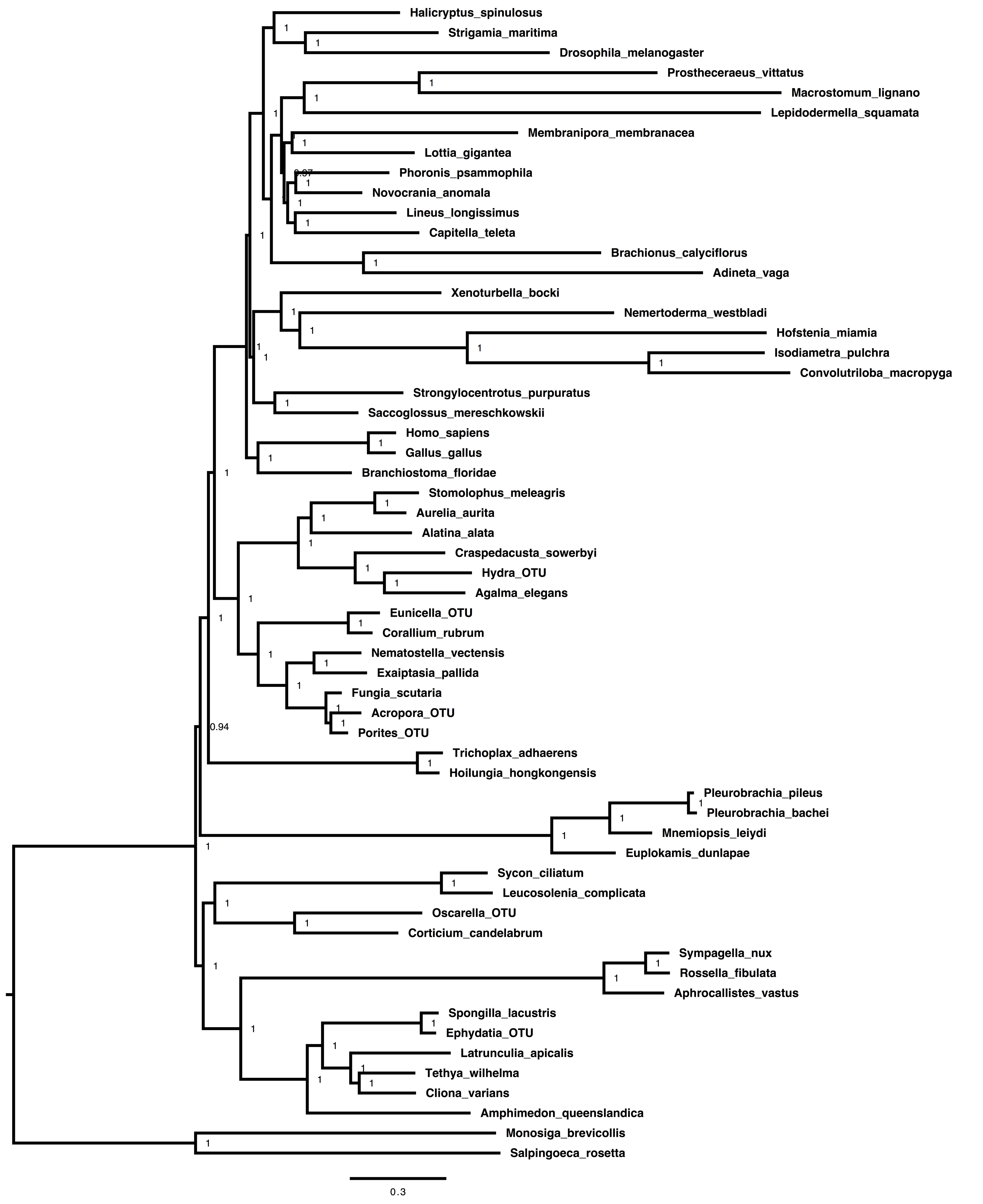

Supplement: S16 Fig — CAT-GTR has been shown to have the best fit to multigene amino acid alignments [68,73]. (TIF) [file pbio.2005359.s016.tif]

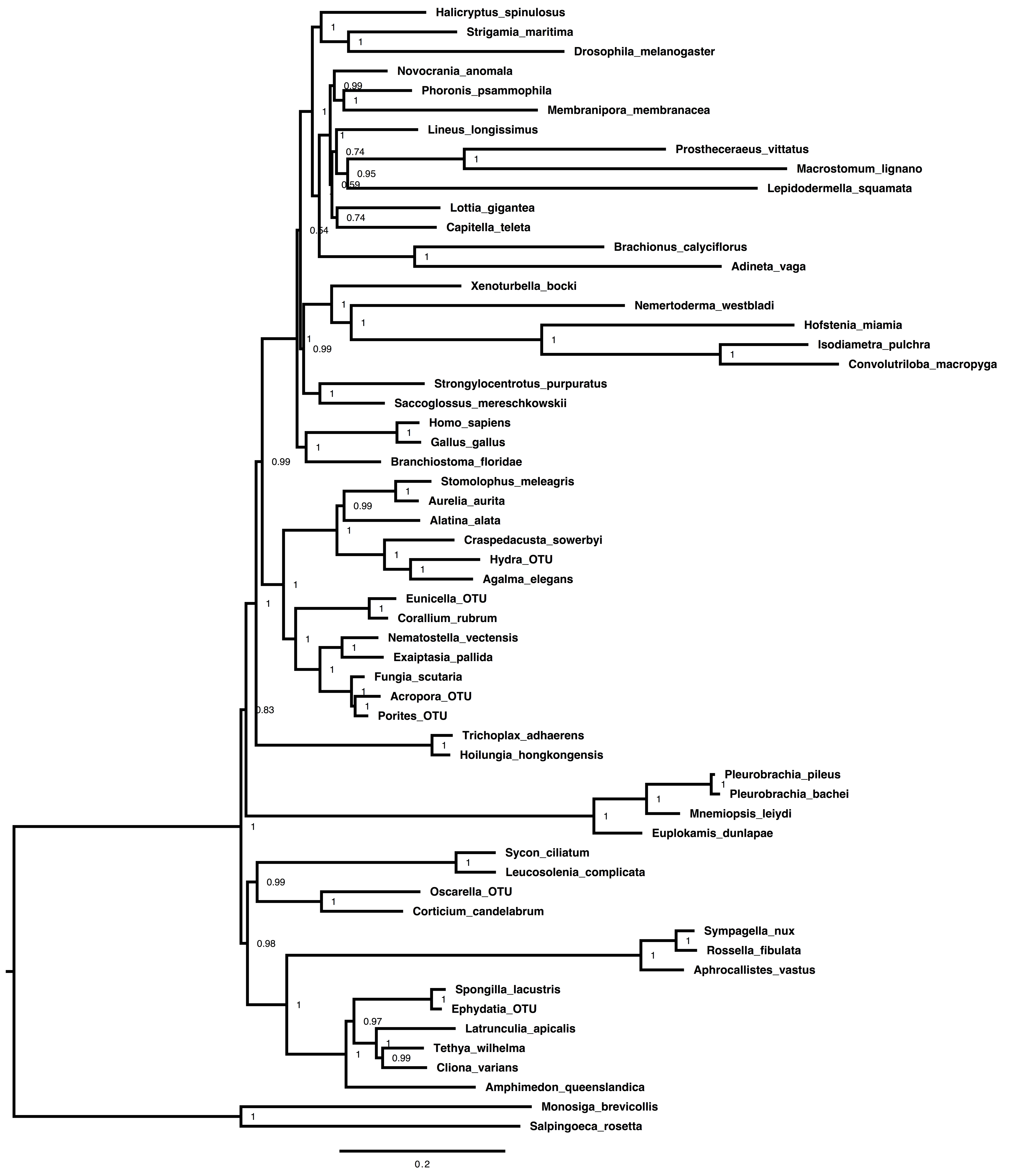

Supplement: S17 Fig — CAT-GTR has been shown to have the best fit to Dayhoff-6 recoded amino acid alignments [68]. Posterior Probabilities are given at nodes. (TIF) [file pbio.2005359.s017.tif]

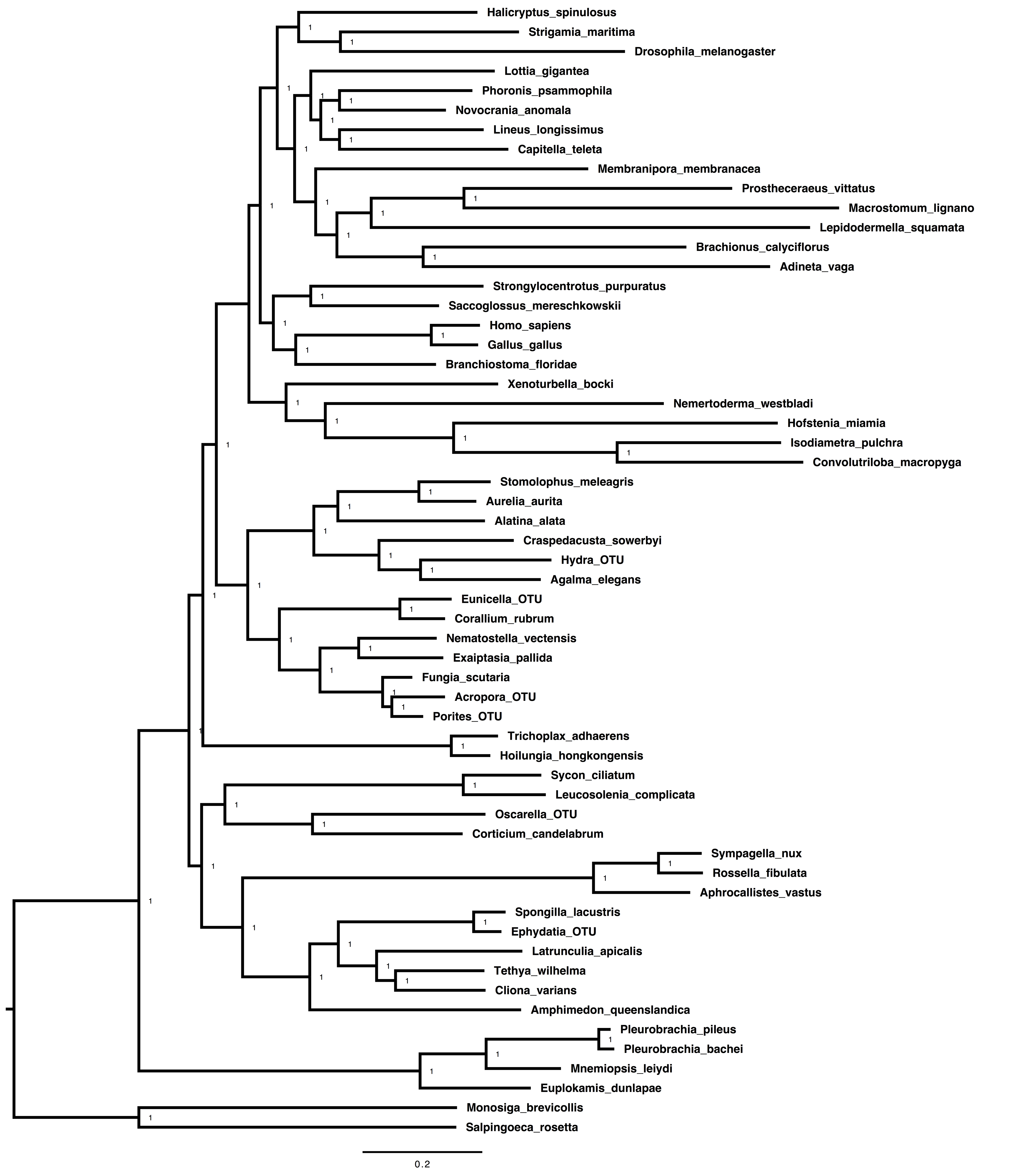

Supplement: S18 Fig — GTR has been shown to have less fit to multigene amino acid alignments compared to CAT-GTR [68,73]. This phylogeny is provided here for comparative purposes only to display the effect of a less fitting evolutionary model on the tree topology (compare to S16 and S17 Figs). Posterior Probabilities are given at nodes. (TIF) [file pbio.2005359.s018.tif]

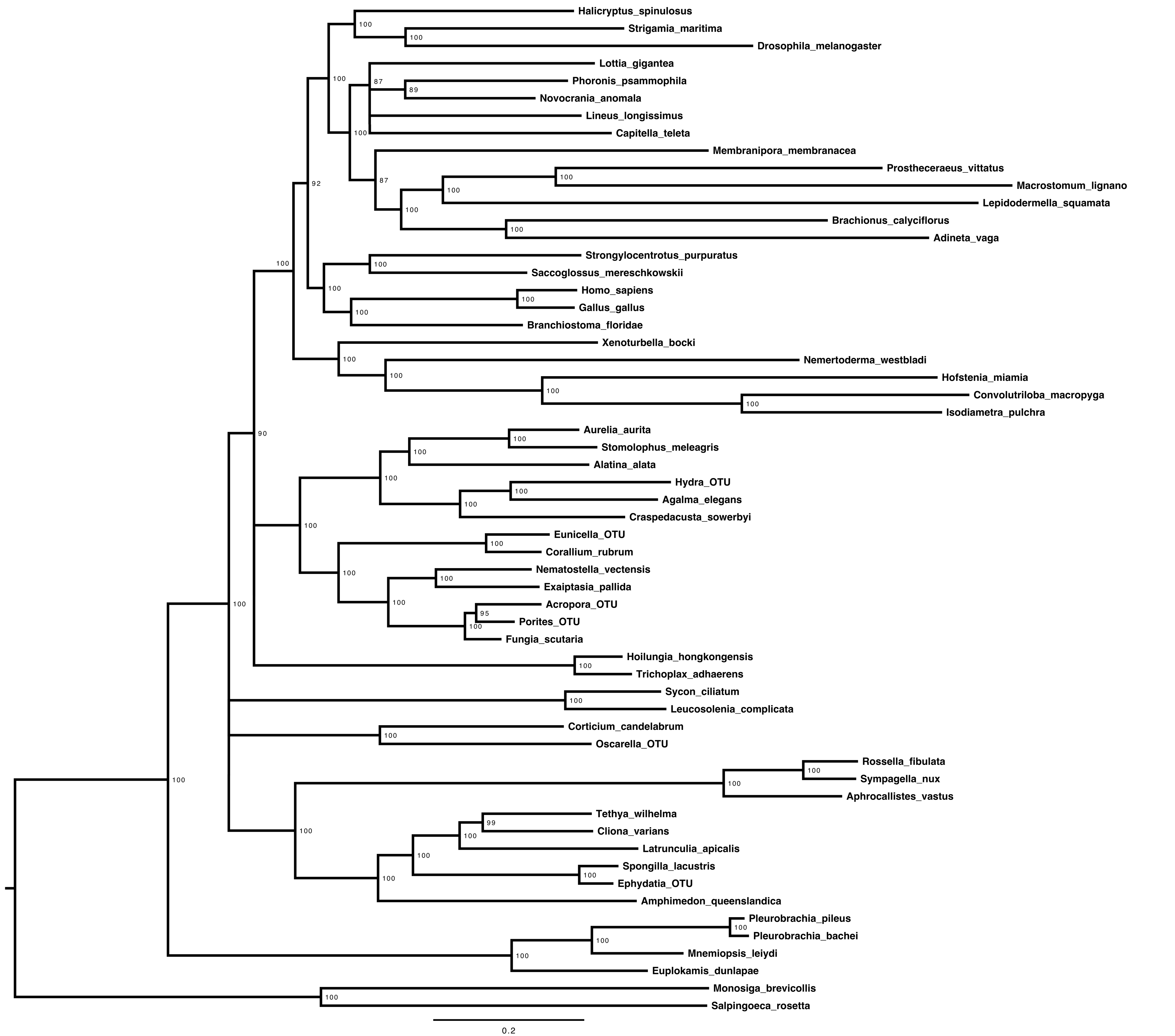

Supplement: S19 Fig — GTR has been shown to have less fit to multigene amino acid alignments compared to CAT-GTR [68,73]. This phylogeny is provided here for comparative purposes only to display the effect of a less fitting evolutionary model on the tree topology (compare to Figs S16 and S17 Figs). Bootstrap support values are given at nodes. Clades with support of <70 have been collapsed and are drawn as a polytomy, due to a lack of confidence in those splits [141]. (TIF) [file pbio.2005359.s019.tif]

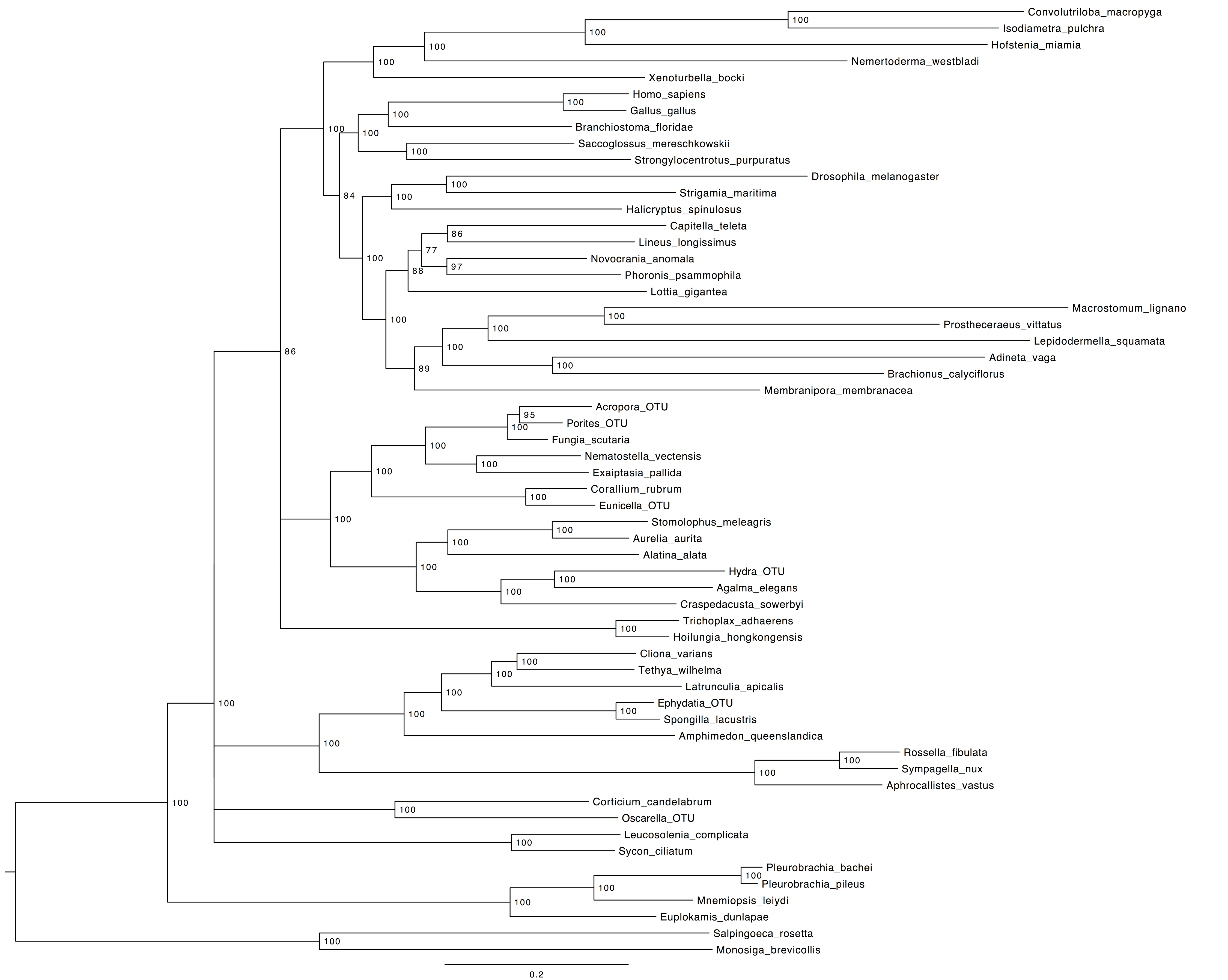

Supplement: S20 Fig — The LG substitution model [141] has been shown to have less fit to multigene amino acid alignments compared to CAT-GTR [68,73]. This phylogeny is provided here for comparative purposes only to display the effect of a less fitting evolutionary model on the tree topology (compare to Figs S16 and S17 Figs). Bootstrap support values are given at nodes. Clades with support of <70 have been collapsed and are drawn as a polytomy because of a lack of confidence in those splits [142]. (TIF) [file pbio.2005359.s020.tif]

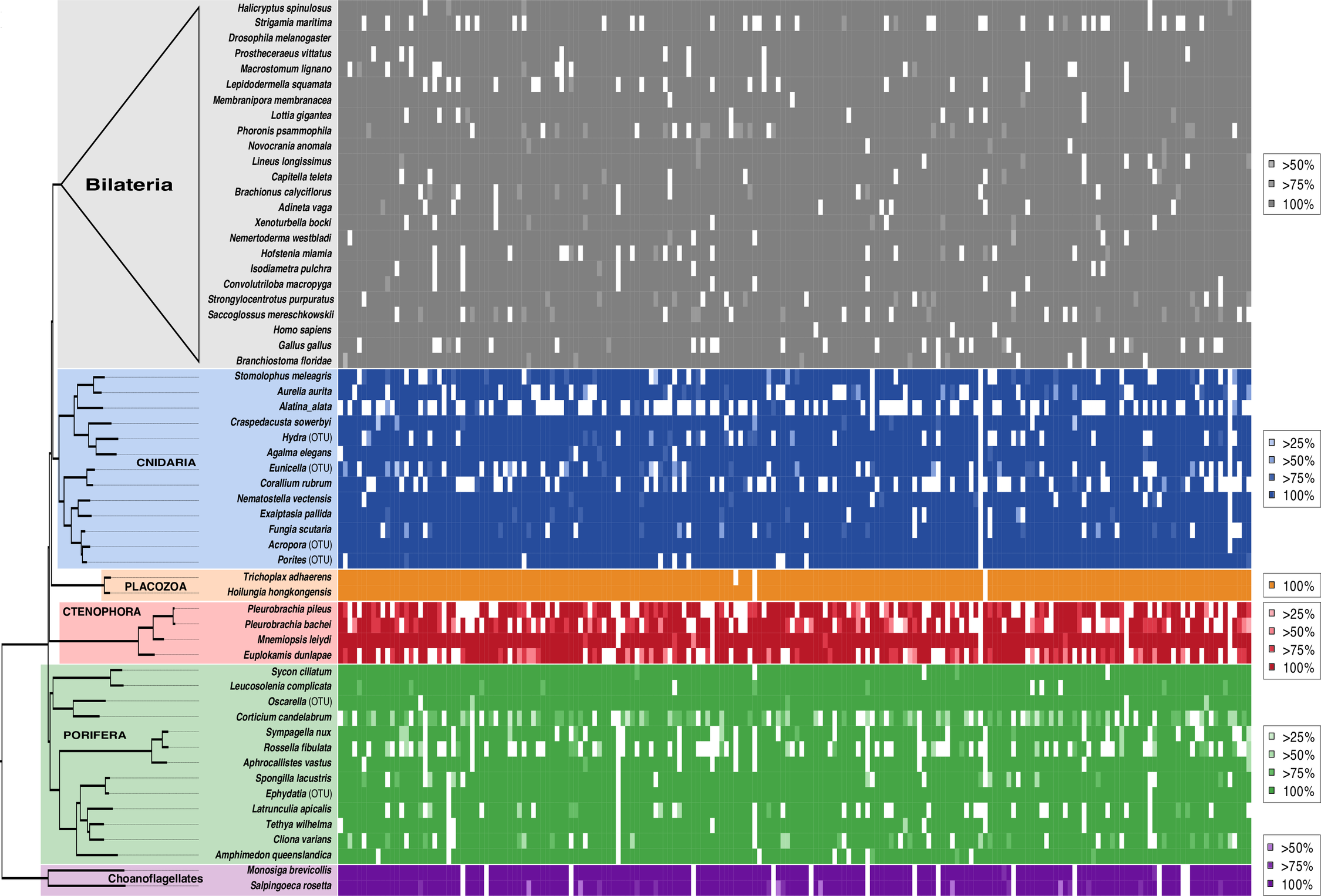

Supplement: S21 Fig — Plotted are all 194 proteins, sorted based on the phylogenetic tree given in Fig 7. White space indicates missing sequence information for a protein. Color intensity is related to the percentage of gene completeness in partial proteins. Note that most of the proteins in the matrix are complete. (TIF) [file pbio.2005359.s021.tif]
